# Methylcytosine oxidation derivatives are preferentially associated with repetitive chromatin and mark active rDNA in *Silene latifolia*

**DOI:** 10.3389/fpls.2026.1918439

**Published:** 2026-09-22

**Authors:** Marcel Hubinský, Václav Bačovský, Pavla Novotná, Radim Cegan, Roman Hobza, José Luis Rodríguez Lorenzo

**Affiliations:** 1 Department of Plant Developmental Genetics, Institute of Biophysics of the Czech Academy of Sciences, Brno, Czechia; 2 CEITEC – Central European Institute of Technology, Masaryk University, Brno, Czechia; 3 National Centre for Biomolecular Research, Faculty of Science, Masaryk University, Brno, Czechia; 4 Department of Experimental Biology, Faculty of Science, Masaryk University, Brno, Czechia

**Keywords:** 5caC, 5fC, DNA methylation, epigenetics, NOR, oxi-mC, rDNA, transposable element

## Abstract

**Introduction:**

DNA methylation and its oxidative derivatives are key components of epigenetic regulation, yet their roles in plant repetitive DNA remain poorly understood. Here, we investigate the genomic distribution of oxidized cytosine modifications: 5-hydroxymethylcytosine (5hmC), 5-formylcytosine (5fC) and 5-carboxycytosine (5caC) within the repeat-rich genome of *Silene latifolia*, a dioecious plant with heteromorphic sex chromosomes.

**Materials and methods:**

Using low-coverage oxi-mC sequencing combined with RepeatExplorer2 clustering, ChIP-seq Mapper analysis, genome mapping, and immunoFISH validation, we identify widespread association of oxi-mCs across major repeat classes, including LTR retrotransposons, DNA transposons, satellite repeats, and ribosomal DNA (rDNA).

**Results:**

While transposable elements largely reflect genomic abundance, satellite DNA shows selective and context-dependent overrepresentation, particularly within the major centromeric repeat clusters (STAR-C), which exhibits association with 5caC in specific chromosome-biased clusters rather than across centromeres globally. In contrast, 45S rDNA arrays display strong and consistent accumulation of oxi-mCs, especially 5fC and 5caC, in transcriptionally active nucleolar organizing regions, supported by both sequencing and cytological evidence.

**Discussion:**

These findings suggest that oxidative cytosine modifications are not randomly distributed but are linked to chromatin state and transcriptional activity, particularly in rDNA loci. Overall, our results reveal cytosine oxidation as a potential additional layer of epigenetic regulation in plant repetitive DNA.

## 1 Introduction

DNA methylation at the C-5 position of cytosine (5-methylcytosine, 5mC) is a key epigenetic modification involved in transposon silencing, genome stability, and gene regulation in plants (Xie et al., 2025). Unlike animals, plant DNA methylation occurs in CG, CHG, and CHH sequence contexts and is tightly linked to the control of repetitive DNA, including transposable elements (TEs) and tandem repeats (Liu and Zhao, 2023). DNA methylation is dynamically regulated through passive and active demethylation. In plants, active demethylation is mediated by DNA glycosylases via base excision repair (Roldan-Arjona et al., 2019). In mammals, an additional pathway involves Ten Eleven Translocases (TET) enzymes that oxidize 5mC into 5-hydroxymethylcytosine (5hmC), 5-formylcytosine (5fC), and 5-carboxycytosine (5caC) (Joshi et al., 2022; Caldwell and Bartolomei, 2023). Among those three oxidative derivatives, 5hmC is so far the most studied modification in mammals (Zheng et al., 2024; Kriukienė et al., 2024). A key mouse brain study mapped 5hmC genome-wide and propose this mark as part of epigenetic regulation of repetitive elements and transposon-derived DNA in the nervous system (Szulwach et al., 2011). During mouse preimplantation development, both 5fC and 5caC exhibited a replication-dependent dilution indicating these marks are stable and the authors proposed a functional role during preimplantation development (Inoue et al., 2011). These oxidized cytosines (oxi-mCs) are also found in fungi. A study on *Coprinopsis cinerea* marked oxi-mCs to DNA transposons, centromeres, and retrotransposons (Chavez et al., 2014), and a recent work on *Laccaria bicolor* concluded that oxi-mCs participate in gene regulation and transposable-element silencing (Licyte et al., 2022). Advances in mass spectrometry analysis have detected oxi-mCs in multiple plant species, including algae, conifers and angiosperms, and dismissed the initial idea that oxi-mC presence in plants was merely residual (Tang et al., 2014; Yakovlev et al., 2019; Viejo et al., 2021; Kalinka et al., 2023). The identification of TET homologues in *C. reinhardtii* (Xue et al., 2019) and recent work in rice linking 5hmC with silent transposons (Wang et al., 2015) and euchromatic regions, including enrichment at ABA-responsive transcription factors in drought stress response (Yan et al., 2025), supports an active role in plant biology. However, their genomic distribution and function, particularly within repetitive DNA, remain poorly understood.

Repetitive DNA constitutes a major fraction of plant genomes and plays essential roles in genome organization and evolution (Kejnovsky et al., 2025; Quadrana and Henderson, 2025). Among them, of particular interest are those transposable elements and satellite repeats components of centromeres (Naish and Henderson, 2024). Centromeres mainly consist of complex arrays of tandem repeats and centrophilic Class I LTR retrotransposons. The combination of centrophilic TEs vs satellites is very variable, and any family from *Gypsy* or *Copia* can be the dominant transposon (Bousios et al., 2025; Horakova et al., 2025). The satellite-transposon association may range from a high abundance of satellite arrays to only centrophilic TE as it has been described in cotton (Chang et al., 2024). Regarding the epigenetic regulation, it is dominated by DNA methylation and siRNA. Histone modifications, such as H3K9me2 in sugar beet, are associated to specific satellite families (Kowar et al., 2016; Naish et al., 2021; Mo et al., 2023). Ribosomal DNA (rDNA) arrays represent another major repetitive component. It is organized in tandem clusters and these clusters are present only in some chromosomes (Roa and Guerra, 2012; Rosato et al., 2016; Volkov et al., 2022). The most interesting feature is the fact that only a subset of copies is transcriptionally active and epigenetically regulated, these copies are organized in NORs (Nucleolar Organizing Regions) (Chandrasekhara et al., 2016; Srivastava et al., 2016; Razzaq et al., 2023). The NORs consist of active rDNA repeat clusters, and they are present in the nucleolus (Pikaard et al., 2023). The active NORs are inherited and the active NORs from one of the parents are also the active ones in the hybrids. This phenomenon, known as nucleolar dominance, also happens between intraspecific crosses and it is not fully understood (Pikaard et al., 2023). The same cluster can contain both active and silent copies, whose regulation reflects a coordinated interplay between 5mC and histone PTMs (Lawrence et al., 2004; Razzaq et al., 2023). Repetitive sequences form a major component of sex chromosomes in dioecious plants, accumulating in non-recombining regions such as the Y chromosome, alongside sex-biased epigenetic asymmetry, referring to differences in epigenetic modifications, such as DNA methylation and histone modifications, between males and females (Cermak et al., 2008; Kejnovsky et al., 2009; Hobza et al., 2015, 2017). The suppression of recombination on the Y chromosome is associated with the accumulation of transposable elements and satellite repeats, which may contribute to genome differentiation between the sexes (Kubat et al., 2008; Kralova et al., 2014; Kubat et al., 2014; Puterova et al., 2018). In *S. latifolia*, the Y chromosome is exceptionally large (~550 Mb) and has been reported to differ from the X chromosome and autosomes in its TE/satellite centromere composition (Moraga et al., 2025). The dioecious plant *Silene latifolia*, with its heteromorphic sex chromosomes and repeat-rich genome, provides an ideal system to study epigenetic regulation of repetitive DNA.

Despite growing evidence for oxi-mCs in plants, their association with specific repeat types such as centromeric satellites, transposable elements, and rDNA remains unclear. In particular, it is unknown whether these modifications are broadly distributed or linked to specific chromatin states or transcriptional activity. Here, we investigate the distribution of 5hmC, 5fC, and 5caC within the repetitive fraction of the *S. latifolia* genome. Using low-coverage oxi-mC sequencing combined with RepeatExplorer2 clustering, comparative analysis, and genome mapping, together with immunoFISH validation, our results show that oxi-mCs are widespread across repetitive DNA but exhibit context-dependent prevalence. While transposable elements largely reflect genomic abundance, satellite repeats display selective associations, and rDNA loci show strong overrepresentation in transcriptionally active clusters. These findings put emphasis on cytosine oxidation as an additional layer of epigenetic regulation in plant repetitive chromatin challenging 5mC-centric models and highlighting dynamic epigenetics in plant sex chromosomes.

## 2 Materials and methods

### 2.1 Plant material

The material used in this study came from the collection of the Institute of Biophysics in Brno, Czech Republic. An inbred population (U17; the crossing of 17 generations of full-sib mating) of male and female *S. latifolia* was used in all the experiments. Plants were grown in a greenhouse under standard long day conditions (22 °C, 16 h light/8 h dark). Vascular leaves post-expansion stage (PO:0001053) were used for nucleic acid isolation and cytological analysis.

### 2.2 Nucleic acid isolation

Total RNA was isolated using NucleoSpin^®^ RNA plant, and genomic DNA was isolated using NucleoSpin^®^ Plant II DNA Kit (Macherey Nagel, Düren, Germany). Three technical replicates using a pool of five individual plants were performed.

### 2.3 Antibody cross reactivity

In order to test the cross-reactivity of the different antibodies, the binary vector pCAMBIA-2301 and the primers F: ACGTAAGGGATGACGCACA and R: CGCGATCCAGACTGAATGCC were used to generate an amplicon of 389 bp. In the PCR, for each reaction, cytidine was replaced with the corresponding modified nucleoside: 5-methyl-2’-deoxycytidine, 5-hydroxy-2’-deoxycytidine, 5-formyl-2’-deoxycytidine, and 5-carboxy-2’-deoxycytidine (TriLink BioTechnologies Inc., San Diego, CA, USA). The reaction was as follows: 10 µl of Buffer 5X, 5 µl of dNTP (10 µM), 1 µl of Q5 High Fidelity DNA Polymerase (New England Biolabs. Inc., Beverly, MA, USA), 2 µl of primers F+R (10 µM each), and 2 µl of DNA (1 ng µl^−1^); ddH_2_O was added to the reaction to bring the final volume up to 50 µl. The program used was: 98 °C for 30 s and 35 cyles at 98 °C for 10 s, 55 °C for 30 s, and 72 °C for 30 s. A final extension step at 72 °C for 2 min was included. The PCR product was purified (Qiagen pcr purification kit) and tested in an agarose gel. A total of 10 ng were used for the antibody detection, mixed with NaOH 0.4 M and EDTA 0.08 mM final concentration in a total volume of 2uL. The probes were denaturated at 95°C for 5 min and immediately placed on ice. For each proble the 2 uL were spotted on a Hybond N^+^ nitrocellulose membrane (GE Healthcare Chicago, Illinois, EE. UU) and air dried for 15 min. The crosslink was performed incubating at 80°C for 30 min. A first wash with PBST (1×Phosphate Buffered Saline plus 0.1% Tween 20) for 10 min was followed by an o/n blocking with 3% BSA in 1×Phosphate Buffered Saline plus 0.1% Tween 20 (PBST) at 4°C. The blocking buffer was removed and the samples were incubated with the appropriate primary antibody (1:1,000) in blocking buffer for 1 h at RT. After two washes with 1xPBST for 15–20 min, the samples were incubated with the secondary antibody, an AP-conjugated anti-rabbit IgG (1:5000) for 1 h at room temperature. The samples were washed 2X with 1xPBST for 15–20 min and visualized using NBT/BCIP (Promega) in an alkaline phosphatase buffer: (0.1 M Tris-HCl, pH 9.5, 0.1M NaCl, 0.05M MgCl_2_). The reaction was stopped washing the membrane in ddH_2_O.

### 2.4 DNA immunoprecipitation assay

The DNA immunoprecipitation (DIP) assay was performed according to Hubinský et al. (2024). A total number of five individual plants for each sex was used for the DNA immunoprecipitation assays. The plants were pooled and three technical replicates for each situation were performed to minimize technical variability. The antibodies used in this protocol were: anti 5mC (Abcam, cat. no. ab214727), anti 5hmC (Abcam, cat. no. ab214728), anti 5fC (Diagenode, cat. no. C15310200), and anti 5caC (Diagenode, cat. no. C15410204-100). A total amount of 20 µg genomic DNA was diluted in 450 μl of 1× IP buffer (10 mM Na_3_PO_4_ pH 7.0, 140 mM NaCl, and 0.05% Triton X-100). A total of seven pulses of 90% intensity for 15 s for each sample at 4 °C were delivered with a UP50H ultrasonic processor (Hielscher Ultrasonics GmbH, Teltow, Germany). An average size of 400 bp and a range of from 200 bp to 800 bp was verified on a 2% agarose gel with. DNA was aliquoted to three 1.5 ml DNA LoBind^®^ Tubes (Eppendorf-5 Prime, Inc., Boulder, CO, USA) corresponding to ± 7 µg of genomic DNA. Samples were heat denatured at 95 °C for 10 min and then immediately cooled on ice for 10 min. A 7 μg aliquot of each corresponding antibody was added and the samples were incubated on a rotating platform overnight at 4 °C. The DNA-antibody complexes were pulled using 50 μl of Invitrogen magnetic Protein G or A Dynabeads and incubated for 2 h on a rotating platform at 4 °C. Samples were washed three times with 1× IP buffer using a magnetic rack and suspended in digestion buffer (50 mM Tris pH 8, 10 mM EDTA, 0.5% SDS) with 1 μl of 20 mg ml^−1^ proteinase K and incubated for 1 h at 50 °C on a rotating platform. Untreated sonicated genomic DNA was processed in parallel and considered as the input sample. Precipitated DNA was recovered using a MinElute PCR Purification Kit (Qiagen, Hilden, Germany). Once the DNA was purified and quantified, we proceeded with Illumina sequencing.

### 2.5 Library preparation and sequencing

The different libraries for DIP-seq were prepared using the NEBNext^®^ Ultra™ II DNA Library Prep Kit. The resulting libraries were low-coverage sequenced using an Illumina MiSeq sequencer, generating 300 bp paired-end reads at the Centre of Plant Structural and Functional Genomics (Olomouc, Czech Republic). At this point, the three replicates were merged for bioinformatic analysis, given that a formal statistical analysis from low pass sequencing might take to misleading conclusions. However, the individual replicates were kept for further correlation analysis among replicates to verify repeatability. For the sequencing of mRNA, we used the services of Novogene (Novogene Corp., Beijing, China). Total RNA samples were sent to the company and they sequenced a paired-end library for each RNA on the Illumina platform NextSeq 2000 (mRNA).

### 2.6 Sequencing data processing

The quality control of sequencing data was carried out using Falco (De Sena Brandine and Smith, 2021). We used the filtering tools available for RepeatExplorer2 and those reads with a quality cutoff Q ≥30 were trimmed to a fixed length of 101 bp. To identify repetitive DNA composition and tandem repeats related to the oxi-mC, these datasets were analyzed using RepeatExplorer2 comparative analysis and Tandem Repeat Analyzer (TAREAN) (Novák et al., 2013, 2017; Neumann et al., 2019; Novák et al., 2020; Novak et al., 2024). We previously run RepeatExplorer2 clustering with default parameters from both, male and female Input samples to create the library. This library was used as reference for the first comparative analysis and for the correlation distance heatmap based on the ChIP-seq Mapper normalized ratio Chip/Input for each individual replicate to test the replicates. For the second comparative analysis, and with the aim to enlarge the variability of repeats to identify, the reference library was created using default parameters for the Input, each ChIPed oxi-mC and both sexes. Clustering and comparative analysis protocols were run using default settings. ChIP-Seq Mapper tool was used to identify and link those enriched annotated clusters to the different oxi-mC. We also run this tool using default settings.

The read mapping and peak calling analysis, as well as the different bioinformatics tools used in this study, were carried out in Galaxy, a public bioinformatics platform (Galaxy, 2024). The reads were trimmed using the tool Trimmomatic, Q ≥ 30 (Bolger et al., 2014). For the mapping of the oxi-mC reads and the significant clusters from ChIP-Seq Mapper in the genome of *S. latifolia* we used BWA-MEM2 (Li and Durbin, 2009; 2010). The ChIPed and Input BAM files were sorted and deduplicated using Picard Tools (Picard Toolkit, 2019), and the resulting files were used as input for the peak caller tool MACS2 (Zhang et al., 2008). The individual replicates were also mapped to the genome and the resulting BAM files were used to track the repetitions using a correlation distance heatmap.

The RNA-seq reads were trimmed using Trim Galore, Q ≥ 30 (Krueger et al., 2023). Trimmed reads were mapped onto the *S: latifolia* V5 reference genome (Moraga et al., 2025) using HISAT with default parameters (Kim et al., 2015). The intersection with the repeat DNA annotation and raw count file generation was carried out using BEDTools and SAMtools (Quinlan, 2014; Danecek et al., 2021).

The contig nucleotide sequence comparison from the rDNA cluster Cl0086 to the 45S rDNA features and cluster Cl0001 to the STAR-C satellite copies from *S. latifolia* genome was performed using VSearch (Rognes et al., 2016).

The sequence for all the STAR-C features from *S. latifolia* genome was aligned using the multiple sequence alignment tool MAFFT (Katoh and Standley, 2013). Following the alignment, we created a phylogenetic tree (Caporaso et al., 2010), and run a parsimony test and a permutation test to compare the STAR-C distribution in our tree from a stochastic distribution. Additionally, we run a clustering analysis using CD-HIT (Fu et al., 2012). The purpose was to identify if in the clustering the features in the same cluster corresponded to the same chromosome or a mix of several chromosomes.

### 2.7 Identification of operational centromere regions

#### 2.7.1 CENH3 antibody design

The gene coding for the CENH3 protein was identified in the genome of *S. latifolia* genome (Moraga et al., 2025) and the NCBI database using the TBLASTN algorithm (Camacho et al., 2009). The isolation of the *SlCENH3* gene, amplification, purification and cloning followed the same protocol as described in Horakova et al. (2025). *SlCENH3* F (AAGTAGCCACCAAGTCATCCG) and R (ATTCTCCGAGCAAGCTCCAG) primers were designed in GENEIOUS PRIME (2023.1.1) (Biomatters Ltd., Auckland, New Zealand) and synthesized in GeneriBiotech (Generi Biotech s.r.o., Hradec Kralové, CZ). The peptide for antibody synthesis was selected based on its hydrophilic profile determined using the KYTE-DOOLITTLE algorithm with linear weight variation model (Kyte and Doolittle, 1982). The histone core domains were determined in HistoneDB 2.0 (Draizen et al., 2016). The antibody against the CENH3 protein of *S. latifolia* was custom raised using the peptide N-RSTPEQPSTSTGKRK-C. Peptide synthesis, rabbit immunization to produce anti-*SlCENH3* antibody, and final peptide affinity purification of antisera were performed by Eurogentec (Kaneka Eurogentec S.A., Seraing, Belgium).

#### 2.7.2 Chromatin immunoprecipitation with sequencing protocol

Nuclei for ChIP experiments were isolated from young leaves of *S. latifolia*. The ChIP-seq protocol with anti-CENH3 antibody was performed as described in Horakova et al. (2025). The sequencing of the libraries was conducted using an Illumina NovaSeq instrument with 150 bp paired-end reads at the Centre of Plant Structural and Functional Genomics in Olomouc, Czech Republic.

#### 2.7.3 ChIP-seq data analysis

The reads from the CENH3 immunoprecipitation and Input libraries were processed using two approaches (Mann et al., 2024). I) We identified the significant repeat clusters using RepeatExplorer2 and following the ChIP-seq mapper protocol (Novák et al., 2013; Neumann et al., 2019; Novák et al., 2020). We identified 4 significant clusters belonging all of them to centromeric repeats (**Supplementary Table S1**). The clusters were mapped using BWA-MEM2 (Li and Durbin, 2009) with default parameters. II) We followed the canonical ChIP-seq analysis to get the broad peaks for CENH3. The reads were mapped after trimming with trimmomatic using BWA-MEM2. The ChIPed and Input BAM files were sorted and deduplicated, and the resulting files were used as input for MACS2 (Zhang et al., 2008). The BED files from both approaches were intersected with the annotations for CRM, STAR-C and STAR-Y. The resulting intervals were extended 500 kb around each repeat and merged if they were <40 kb apart. Large clusters of STAR-C are present not only in the centromere but also in the distal part of chromosomes 9 and Y. This fact involved CENH3 signal in these regions, providing false positive regions, as STAR-C presence in A9 and Y chromosome may originate from chromosomal rearrangement. A final output with the top 3 candidate intervals was used to visually determine the centromere region using IGV (Thorvaldsdóttir et al., 2012). We considered the delimited regions after this analysis were valid to compare the centromeric repeats inside and outside the centromere region.

### 2.8 Cytogenetics

#### 2.8.1 ImmunoFISH

Vegetative leaves from male and female *S. latifolia* plants were fixed using paraformaldehyde 4% in 1x PBS. Interphase nuclei were isolated chopping the tissue in lysis Buffer (LB01: 15 mM Tris, 2 mM Na_2_EDTA, 0.5 M Spermine, 80 mM KCl, 20 mM NaCl, 15 mM β-mercaptoethanol, 0.1% (v/v) Triton X-100, pH 7.5) and filtrating the lysate through a 30 µm mesh filter. Isolated nuclei were dispersed on the slides using cytospin at 600 rpm. Samples were denatured 8 min. in 200 mM NaOH diluted in 70% ethanol and washed for 5 min. in 1xPBS. The blocking was done with 3% BSA, 0.1% Tween 20 in 1X PBS for 1 h at room temperature. After the blocking, we proceeded with the primary antibody incubation diluted 1:400 in 1% BSA, 0.1% Tween 20 in 1X PBS overnight, using antibodies against 5mC (Abcam, cat. no. ab214727), 5hmC (Abcam, cat. no. ab214728), 5fC (Diagenode, cat. no. C15310200), and 5caC (Diagenode, cat. no. C15410204-100). After primary antibody incubation, the samples were washed 3 times in 1xPBS for 5 min, and incubated with the secondary antibody conjugated with Cy3 fluorescein (goat α-rabbit; AB_2313593), diluted 1:400 in 1% BSA, 0.1% Tween 20 in 1X PBS for O/N. After washing 3 times in 1x PBS for 5 min, we continued with the FISH protocol according to Bačovský et al. (2019). DNA probes for 5S and 18S rDNA were prepared as described in Horakova et al. (2025). STAR-C probe preparation was performed as described in Bačovský et al. (2019), following the same protocol for Nick translation and purification. The samples were dried in ethanol series and the nuclei were counterstained with DAPI (4’,6-diamidino-2-phenylindole) in Vectashield Antifade Mounting Medium. A negative control without the primary antibody incubation was also included in this work (**Supplementary Figure S1**). Images were captured with an inverted microscope IX85 (Evident, Japan) equipped with UPLXAPO oil-immersion objectives (40×, NA 1.40; 60×, NA 1.42; 100×, NA 1.45), filter sets for excitation wavelengths of 405, 490, 568, 649, and 779 nm, a monochrome Hamamatsu ORCA-Flash4.0 camera (Hamamatsu Photonics K.K., Japan), LED illuminator (IX5-LHLEDB), and an Excelitas X-Cite XYLIS II light source.

#### 2.8.2 AgNOR

The nuclei were processed using the same protocol we used for immunoFISH. The silver staining was done according to (Huang et al., 2012) using 2% gelatin, 1% Formic acid and 50% (w/v) AgNO3 for 10, 15 and 20 min. Samples were washed with H_2_O and mounted with DAPI in Vectashield Antifade Mounting Medium. Images were taken using an IX85 inverted microscope as previously indicated and a color camera DP23-CU-1-2 (Evident).

#### 2.8.3 oxi-mC fluorescence quantification and colocalization analysis

The mean fluorescence for the antibody and DAPI signals were used to calculate the relative fluorescence as follows: Antibody mean intensity/DAPI mean intensity. A linear region of interest (ROI) across the nuclei including the nucleoli was used to define the nucleoli based on the DAPI signal decrease using Fiji (Schindelin et al., 2012). The plugin color profiler was used to split the RGB channels and calculate both the mean values for the global image analysis and the data for the graphical signal distribution in the nucleoli. The colocalization test between STAR-C/5caC and 45S/5fC-5caC were performed using the colocalization tool from Fiji (Schindelin et al., 2012). We calculated the Maximum Truncated Kendall Tau (MTKT): An advanced localized spatial rank-correlation metric designed to handle spatial heterogeneity and background noise by applying thresholds to Kendall’s tau calculations. This parameter evaluates tail-dependencies and robust local pixel correlations while ignoring uninformative background noise intensities.

### 2.9 Data analysis and plotting

Processed sequencing data were analyzed using R statistics software with regular scripts adapted to our data (R Core Team, 2025).

The statistical analysis for the RE2 y BAM files included a Pearson`s correlation distance heatmap based on the ChIP-seq Mapper normalized ratio Chip/Input for each individual replicate and a correlation distance heatmap based on the BAM files after mapping the reads in the *S. latifolia* genome.

The 45S rDNA cytogenetics statistical analysis included a test for homocedasticity, and once met, the parametric test ANOVA followed by a Tukey test (p ≤ 0.05). The data for this analysis were the mean values of the antibody signal/DAPI signal ratio. Six replicates were used combining male and female nuclei.

For the statistical analysis of 45S rDNA sequencing data, we calculated the probability of observing the number of summits actually observed in that feature, given the total number of fC summits in that replicate. or each feature, observed 5fC and 5caC summit counts were compared with length-based expected counts, and enrichment significance was evaluated using Fisher’s exact test. P-values were adjusted for multiple testing using the Benjamini–Hochberg procedure, and features with an FDR-adjusted *p*-value < 0.05 were considered significant. mC and hmC quantification showed little or no feature-level enrichment. Independently, normalized male and female mRNA signal across the same 45S features was used to identify transcriptionally enriched features based on z-scores. These analyses allowed identification of individual 45S units showing significant 5fC/5caC enrichment and assessment of their correspondence with potentially transcriptionally active rDNA features. The following packages were used for data manipulation and plotting: Bioconductor packages (Huber et al., 2015), dplyr (Wickham et al., 2023), tidyr (Wickham et al., 2024) and ggplot2 (Wickham, 2016). ChatGPT was also used as support for the design of the scripts (OpenAI, 2025).

## 3 Results

In this study, we investigated the genomic distribution of oxidative cytosine modifications within the repetitive DNA fraction of the *S. latifolia* genome. Using low-coverage oxi-mC sequencing combined with RepeatExplorer2 clustering and genome mapping approaches, we identified multiple repeat families associated with different cytosine oxidation states. Our RepeatExplorer2-based analysis of low-pass oxi-mC sequencing data reveals that oxidative cytosine derivatives (5hmC, 5fC and 5caC) are broadly distributed across the repetitive fraction of the *S. latifolia* genome. By combining comparative clustering and ChIP-seq Mapper approaches, we identified modification-specific patterns across satellite DNA, LTR retrotransposons and rDNA, highlighting a previously underexplored layer of epigenetic complexity in plant repetitive chromatin. The dot-blot analysis showed no detectable cross-reactivity among the four antibodies under the conditions tested, supporting their specificity and providing additional confidence in the antibody-based results presented in this study (**Supplementary Figure S2**). We additionally assessed the reproducibility of the technical replicates using Pearson correlation analyses based on both the ChIP-seq Mapper normalized ChIP/Input ratios and the BAM files after mapping to the *S. latifolia* reference genome (**Supplementary Figures S3**, **S4** and **Supplementary Table S2**). Within-condition correlations were generally high, although their magnitude varied among modifications and replicates. For the mapped BAM data, mean within-condition Pearson correlation coefficients ranged from 0.785 for male 5caC to 0.982 for male input samples, with most conditions showing mean correlations above 0.83. The lowest individual within-condition correlation was observed between CCM1 and CCM2 (r = 0.694), while these replicates showed higher correlation with CCM3 (r = 0.762 and r = 0.898, respectively). Thus, although technical replicate concordance was not uniform across all conditions, the replicates generally showed substantial similarity and no replicate displayed a consistently discordant pattern. Because the three replicates were generated from the same pool of five individual plants and therefore represent technical rather than independent biological replicates, they were merged to increase sequencing depth and provide a more comprehensive representation of the pooled material for downstream analyses.

### 3.1 Repeat cluster and comparative analysis

The comparative analyses from both, gDNA and oxi-mC libraries, evidenced differences between Input and oxi-mC, mainly in satellite and tandem repeat DNA clusters and clusters related to Class I LTR retrotransposons (**Figure 1A**). An increasing number of Class I LTR families is observed compared to the first comparative analysis (**Figure 1B**), especially for 5mC and 5hmC. The satellites, on the other hand, are split into two blocks and are mainly localized in 5fC and 5caC. Differences can also be observed comparing males and females for each oxi-mC in the mentioned repeats.

**Figure 1:**
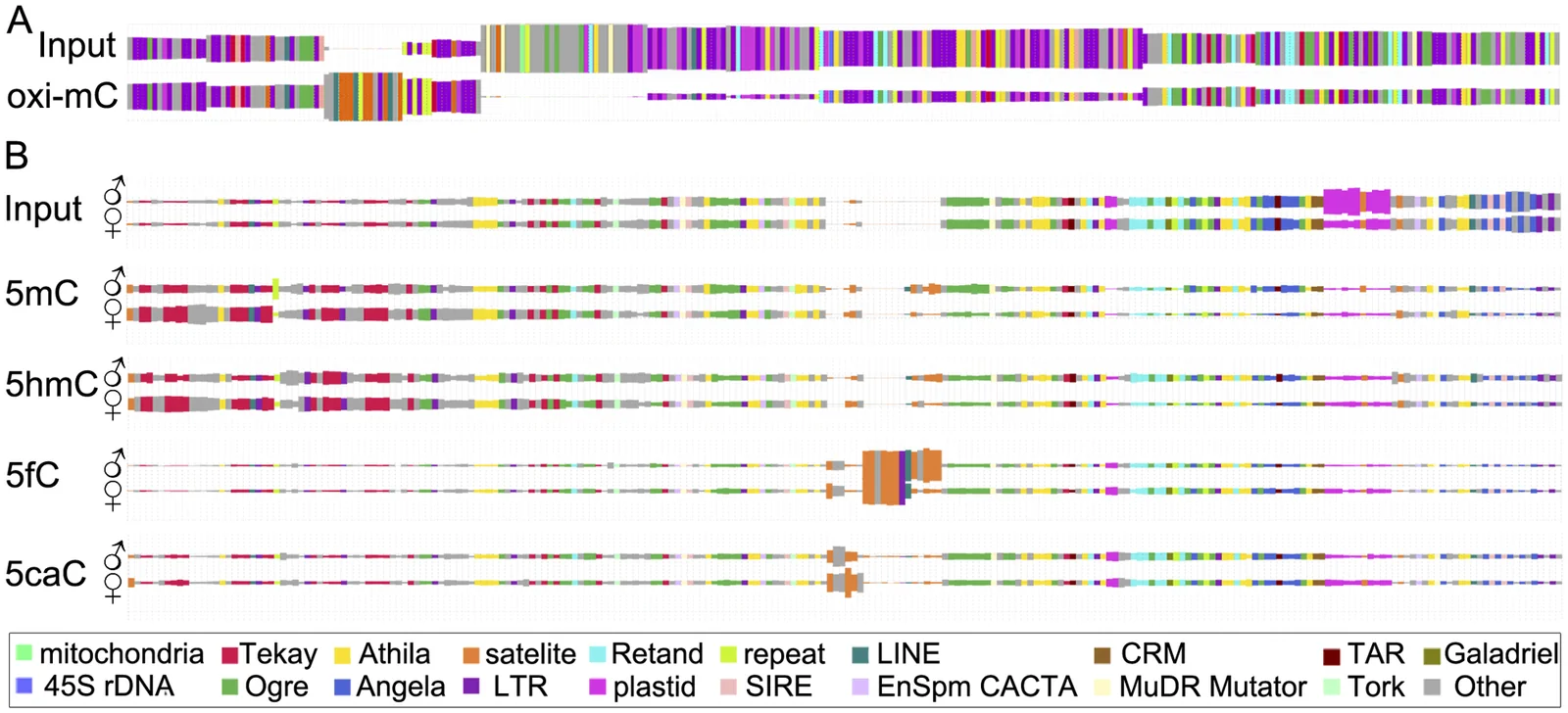
**(A)** Automatic annotation from RepeatExplorer2 comparative analysis oxi-mC/Input using a repeat library from *S. latifolia* male and female gDNA reads. Based on the bar high, satellite DNA and simple repeats represent the main difference followed by unknown LTR retrotransposons. **(B)** Automatic annotation from RepeatExplorer2 comparative analysis. Each different cytosine modification/sex combination was considered as an independent population for the cluster library generation. The differences among the cytosine modifications are evident for LTR retrotransposons compared to Input. An increasing number of Class I LTR families is observed compared to the first comparative analysis, especially for 5mC and 5hmC. The satellites, on the other hand, are split in two blocks and are mainly localized in 5fC and 5caC. Differences can also be observed comparing male and females for each oxi-mC.

In order to identify the relevant clusters, the ChIP-seq Mapper tool protocol (Neumann et al., 2012) was applied to our oxi-mC sequencing data using the gDNA cluster library as a reference. The significant clusters supported the main differences from the comparative analysis (detailed in **Supplementary Table S3**). The meaningful clusters from ChIP-seq Mapper considering each cytosine modification and sex as individual population were identified (**Supplementary Table S4**). A manual annotation of the clusters was carried out based on the protein database annotation from RepeatExplorer2 and the repeat annotations from Moraga et al. (2025). We mapped and annotated the clusters from both analyses (**Supplementary Figures S5**, **S6**). We associated the different repeats with the oxi-mC and observed the modifications present in all repeat types: LTR retrotransposons, DNA transposons and satellite/tandem repeats. The main difference was found on 5caC, the only modification differentially present in the centromeric satellite STAR-C (hereafter referred to STAR-C), which is the major tandem repeat forming the S. latifolia chromosome centromeres. The differences among the cytosine modifications are more evident for LTR/unknown compared to Input. The analyzed satellites are differentiated in two blocks suggesting two different groups. We observed an increasing number of Class I LTR families and a few clusters annotated for Class I LTR transposon CRM (Centromere Repeat Maize), previously described as *Sil1* and *Sil2* (Macas et al., 2011).

### 3.2 Centromere region

The centromeres of autosomes and sex chromosomes, except the Y, are composed of the centrophilic transposon CRM and STAR-C. The Y chromosome centromere possesses dominantly centrophilic CRM. Additionally, STAR-C can be found in chromosomes 9 and Y present in two large clusters in the distal arm of both chromosomes. This fact posed the question if 5caC was more frequently observed in the centromeric region for STAR-C or, on the other hand, STAR-C is associated with 5caC genome wide or specific arrays. To answer it, we defined three approaches. First, we delimited the operational centromere regions (**Supplementary Figure S7**), and calculated the percentage of features covered for STAR-C and centrophilic CRM clusters mapped in our centromere region compared to the percentage of features covered for STAR-C and centrophilic CRM outside this region (**Figure 2A**). This first approach did not suggest any bias toward the centromere for any oxi-mC or sex. Second, we detected 5caC and STAR-C on somatic nuclei by immunoFISH. Overall, STAR-C showed low, positive, non-random colocalization correlation (**Figure 2B**). A partial overlap can be observed for one cluster in a representative nuclei (**Figure 2C**). This result supports the first approach and indicates 5caC has a connection with STAR-C but it is not enriched genome wide, only in some clusters. The last approach was based on the sequence similarity from all the STAR-C features (**Figure 2D**). We performed a multiple sequence alignment and made a phylogenetic tree. To answer the question whether sequences from the same chromosome are significantly clustered on the tree, we run a parsimony test and a permutation test (**Supplementary Figure S8A**). The parsimony score was 23465 and the p-value for the permutation was significant p < 0.001, which means that none of the 1000 random permutations produced a score as low as the observed one for our tree. The sequence similarity from all the STAR-C features was examined by clustering the identical sequences and inspecting the presence of one or more than one chromosome in the cluster. More than half of the clusters are completely chromosome-specific and even when mixed (**Supplementary Figure S8A**), clusters are still strongly biased toward one chromosome. The biological meaning of these results is STAR-C repeats are not randomly distributed and they show chromosome-specific similarity. A representation of cluster Cl0001 corresponding to centromeric satellite STAR-C from the comparative analysis reveals the enrichment in 5caC (**Supplementary Figure S8B**). Having this in mind, we calculated the similarity of the contigs from cluster Cl0001, and enriched for 5caC according to ChIP-seq mapper, with the sequence from all STAR-C features present in the genome of *S. latifolia*. Given the connection of identical STAR-C features present in the same chromosome, we filtered those hits with a 100% similarity and calculated their chromosome distribution. The distribution of STAR-C features in the genome (**Supplementary Figure S8C**) did not correspond to the distribution of cluster Cl0001 (**Supplementary Figure S8D**). This result revealed cluster Cl0001, enriched in 5caC, is a subpopulation from the whole STAR-C sequence population and this cluster was mainly present in chromosome 1. This last approach supported the enrichment in specific clusters present in concrete chromosomes observed in the partial colocalization of STAR-C and 5caC. According to the mRNA-seq analysis all STAR-C copies displayed no read counts.

**Figure 2:**
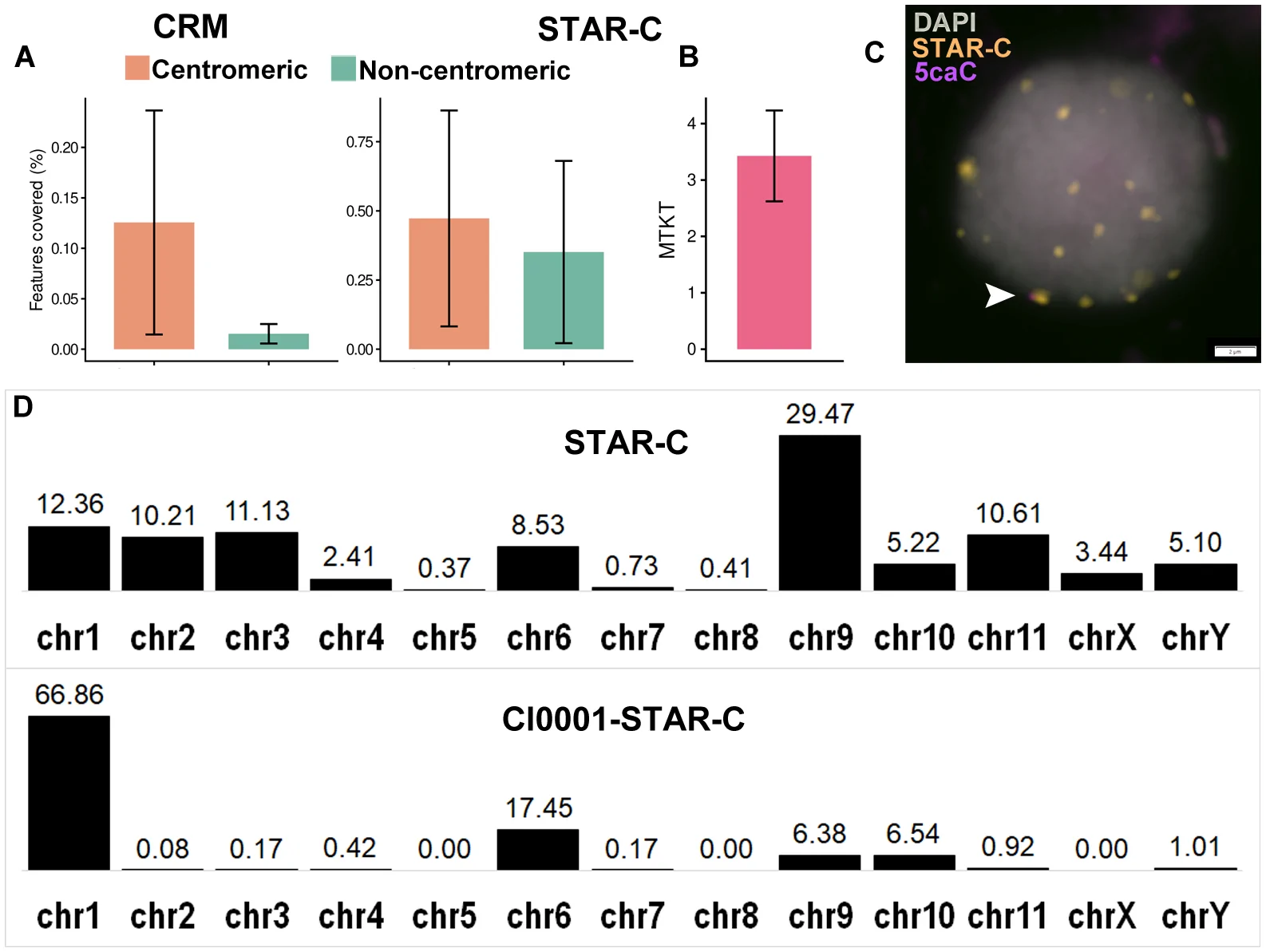
Detection of centromere related repeats and 5caC in *S*. *latifolia*. **(A)** Centromeric vs Non-centromeric STAR-C and CRM percentage of covered features. In general, and based on these two main repeats, we cannot conclude bias to centromeres. **(B)** Maximum Truncated Kendall Tau (MTKT) rank correlation for STAR-C and 5caC colocalization. Positive low values indicate a low not random correlation **(C)** ImmunoFISH on a representative *S. latifolia* nucleus with localized 5caC (magenta) and STAR-C (yellow). Nuclei are counterstained in DAPI (gray). An overlap in a few STAR-C clusters and 5caC signal is observed in both sexes. **(D)** Top, normalized (number of STAR-C features per chromosome/total number of genome centromeric satellite STAR-C features)*100 STAR-C distribution per chromosome, chromosome 9 shows the largest percentage in the genome. Bottom, normalized (number of STAR-C features identical to Cl0001 per chromosome/total number of STAR-C features identical to Cl0001)*100 STAR-C distribution per chromosome. A percentage of 66.86% of features were present in chromosome 1, higher than expected by random distribution and also different from STAR-C chromosomic distribution in *S. latifolia* genome. Scale bar= 2µm.

### 3.3 rDNA

The presence of 45S rDNA in the comparative analysis prompted us to map the reads in the genome of *S. latifolia*. We calculated the normalized feature coverage for all the families and 45S rDNA exhibited a percentage equal or higher than 20% in most of the cytosine oxidative modifications; 5hmC, 5fC and 5caC in females and 5fC in males (**Supplementary Figure S9**). We considered this repeat for further analysis because 45S rDNA is distributed in discrete clusters and only few of these clusters are active in plant genomes. In *S. latifolia* there are 115 copies along the genome, they are not present in all the chromosomes and, if present, they vary in number (**Figure 3B**). A total of 49.2% of the individual clusters was covered by at least one summit (a single base reduction from the narrow peaks intervals) from the different oxi-mCs combining both sexes. This coverage was mainly focused on those potential active clusters according to the RNA data in chromosomes 2 and 9, and in a lesser extent in chromosomes 1 and 6 (**Figure 3A**). The percentage of copies covered by the oxi-mC in the individual clusters ranged from 47.8% in chromosome 1 to 86.1% in chromosome 9. For the individual feature intervals, those potentially active and enriched loci for 5fC and 5caC corresponded to chromosomes 2 and 6 (**Figure 3C**).

**Figure 3:**
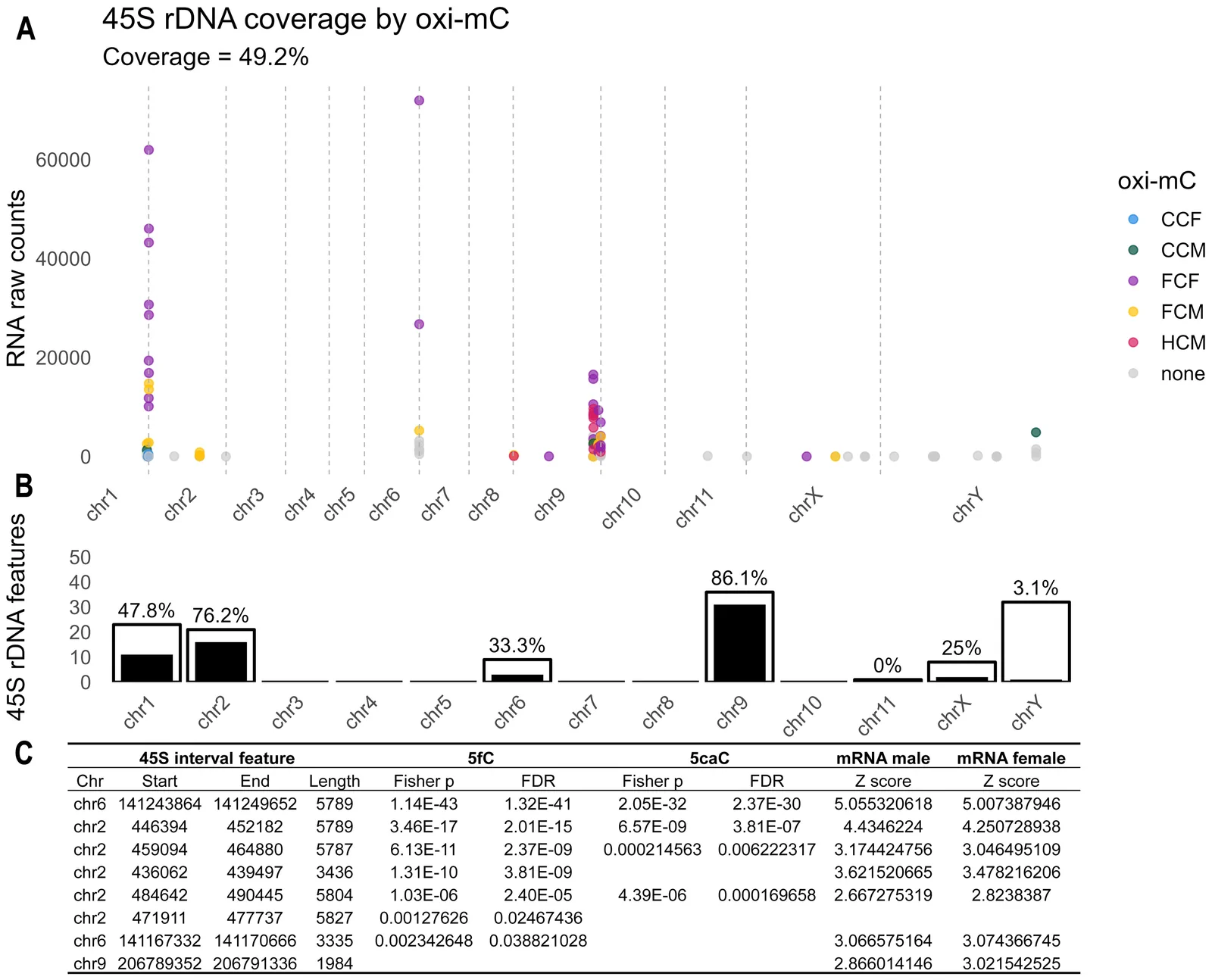
Distribution of individual oxidative modifications within the genome of *S*. *latifolia*. **(A)** Scatter plot mapping of 45S rDNA feature expression intersected with oxi-mC summits along *S. latifolia* chromosomes. Colored dots based on different oxi-mCs and sex mainly gather around active 45S rDNA clusters in chromosomes 2 and 9, and in a lesser extent in chromosomes 1 and 6. **(B)** Bar plot indicating the number of 45S rDNA clusters and distribution in *S. latifolia* chromosomes. Bar filling and percentages correspond to the features covered by at least one summit from the different oxi-mCs and sex. **(C)** Summit positions from six replicates per DNA modification (male and female combined) were intersected with 45S rDNA features using single-base summit coordinates. For each feature, observed 5fC and 5caC summit counts were compared with length-based expected counts, and enrichment significance was evaluated using Fisher’s exact test. P-values were adjusted for multiple testing using the Benjamini–Hochberg procedure, and features with an FDR-adjusted *p*-value < 0.05. Normalized male and female mRNA signal across the same 45S features was used to identify potential transcriptionally enriched features based on z-scores. The clusters with potential enrichment and/or active expression belonged to chromosomes 2, 6 and 9. CCF-5caC female, CCM-5caC male, FCF-5fC female, FCM-5fC male, HCM-5hmC male.

The RE2 comparative analysis annotation of cluster Cl0086 belongs to 45S rDNA. A detailed analysis revealed 5caC as the predominant mark in the different protein domain annotations of the 45S rDNA (**Supplementary Figure S10**). Based on the sequence of the different contigs from Cl0086, we carried out a nucleotide alignment and calculated the percentage of identity compared to the sequence of the 115 features along the male and female genome of *S. latifolia*. Those contigs with similarity over 90% matched feature intervals from chromosomes 2, 6, 9 and Y for males. A large number of raw counts was observed for most of those intervals corroborating the read mapping (**Supplementary Figure S11**). Twelve contigs from the one hundred integrating Cl0086 represented the combination between 5caC and the main active 45S rDNA clusters.

Additionally, we calculated the raw mRNA counts in those features covered by the different oxi-mC in the tandem repeats including the satellite TR1 (**Supplementary Figure S12A**). The reason was this satellite colocalizes with 45S rDNA in some chromosomes, both in active and silent clusters. We found out the satellite TR1 has a large number of features with high mRNA counts for 5fC and 5caC, and they are present in the potential active rDNA clusters (**Supplementary Figure S12B**).

The low depth sequencing represents a partial view of the whole genome. To support the trend suggested by the sequencing analysis and be able to compare all the clusters in both sexes, we carried out an immunoFISH on male and female *S. latifolia* nuclei. We combined the antibody detection for each cytosine modification with physical localization of 5S and 45S rDNA. First, we identified the Nucleolar Organizing Region (NOR) and the associated 45S rDNA active clusters staining *S. latifolia* nuclei with silver nitrate (AgNOR) (**Supplementary Figure S13**). A colocalization between the dark brown stained nucleolus and the hollow circle in the blue nucleus was observed and allowed us to use DAPI staining to confine the nucleolus area inside the nucleus in the immunoFISH. Darker brown dots identified in the perimeter of the nucleolus indicated the active 45S rDNA clusters. We observed a colocalization among few 5S rDNA clusters and the different cytosine modifications, but no trend or specific modification associated to individual loci (**Supplementary Figure S14**). Compared to 5S rDNA, from two to three 45S rDNA clusters were present in the nucleolus denoted by a white arrow (**Figure 4**). These clusters exhibited a clear colocalization with 5fC and 5caC (**Supplementary Figure S15**). Additionally, we observed an increasing accumulation in the nucleolus along with the progressive 5mC oxidation derivatives 5hmC, 5fC and 5caC in both sexes. This increase was supported by the mean relative fluorescence present in the nucleoli. A significant higher fluorescence was found for 5fC and 5caC in both sexes (**Figure 4**).

**Figure 4:**
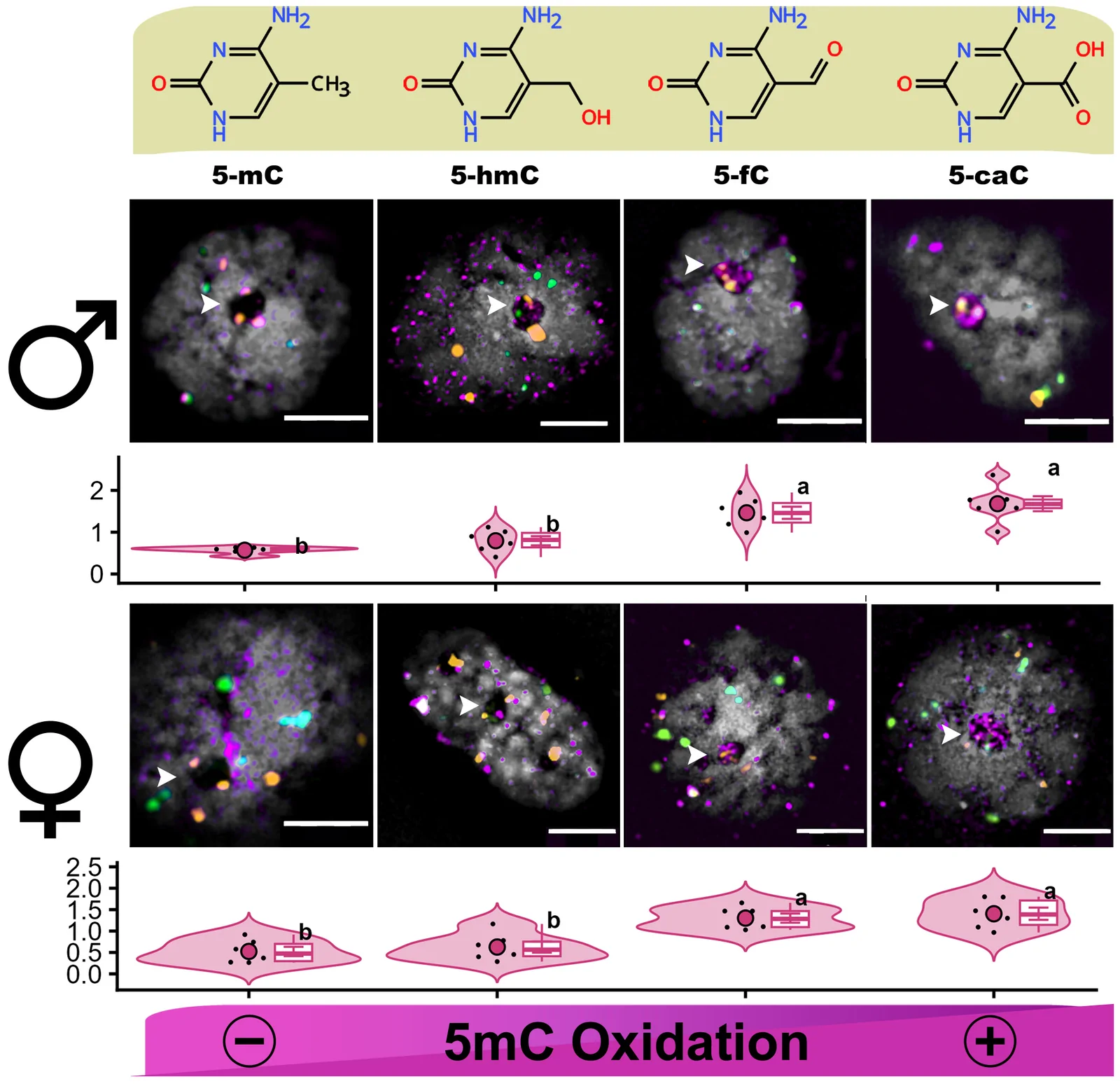
Localization of individual 5mC oxidative derivatives on male and female *S. latifolia* nuclei. An increasing signal concentration is observed in the nucleolus for the individual cytosine residues (magenta), localized together with 45S rDNA (yellow) and 5S rDNA (green). Nuclei counterstained in DAPI (gray). The white arrow points to the nucleolar organizing region. The two active rDNA 45S clusters present in the nucleolus overlap their signal with 5fC and 5caC enrichment. Scale bar= 5µm. The plots represent relative mean fluorescence (antibody signal/DAPI signal ratio) for each modification in the selected region of the nucleolus. Both sexes displayed significant differences between 5mC and 5hmC compared to 5fC and 5caC. The parametric test ANOVA followed by a Tukey test (p ≤ 0.05) were used to define statistical differences (n = 6).

## 4 Discussion

### 4.1 Oxidative cytosine modifications are broadly associated with repetitive DNA

The results indicate that oxi-mCs are widely distributed across diverse repetitive elements including satellites, tandem repeats, DNA transposons, and LTR retrotransposons. The prevalence of oxi-mC across many repeat clusters is consistent with findings in rice and several animal species (Wang et al., 2015; Deniz et al., 2019). In plants, DNA methylation dynamics are tightly linked to the regulation of transposable elements and repetitive DNA (Liu and Zhao, 2023). Although the mechanisms generating oxidized cytosine derivatives in plants remain elusive, several studies have reported the presence of these modifications and indicate that they may contribute to epigenetic plasticity in plant genomes (Wang et al., 2015; Yakovlev et al., 2019; Viejo et al., 2021; Kalinka et al., 2023; Hubinský et al., 2024; Yan et al., 2025). Our results reinforce this hypothesis by demonstrating that multiple repeat families harbor oxi-mC signals in *S. latifolia*. We observed sex-specific differences, with female samples showing a broader representation of oxi-mC-associated clusters. Given that *S. latifolia* is a dioecious species with heteromorphic sex chromosomes, this may reflect differences in chromatin organization or repeat composition between sexes, as previously indicated for plant sex chromosomes (Cermak et al., 2008; Bačovský et al., 2019; Rodríguez Lorenzo et al., 2020).

### 4.2 Satellite DNA and centromeric repeats: selective rather than global association

The association of 5caC with STAR-C, a major component of *S. latifolia* centromeres together with CRM retrotransposons, consistently identified among significant clusters, supported their relevance in oxi-mC-enriched datasets. However, further analyses indicate that this relationship is not straightforward. Quantitative comparison between centromeric and non-centromeric regions did not reveal enrichment of oxi-mCs in centromeres. Additionally, immunoFISH experiments showed only sporadic colocalization between 5caC and STAR-C arrays in both sexes and finally, the STAR-C cluster alignment was chromosome biased. Together, these results indicate that 5caC is associated with specific STAR-C clusters but is not a universal feature of centromeric DNA. STAR-C cluster identity indicated *S. latifolia* chromosomes have their own superclusters sharing a small percentage with other chromosomes. This observation is consistent with current models of plant centromere organization. In *Arabidopsis* each chromosome has a private library of CEN180 monomers forming the centromere, which are in a different evolutionary stage (Naish et al., 2021). Considering this chromosome-cluster distribution, the accumulation of Cl0001 corresponding to STAR-C in chromosome 1, and in less extent in chromosome 6, suggest a connection between 5caC and these chromosomes. The centromere drive hypothesis proposes that tandem repeat arrays play a key role in recruiting the CENH3 protein. However, the gradual accumulation of transposable elements can become deleterious, leading to periodic elimination. As a result, centromeres are typically enriched for younger transposable elements, while older insertions are removed through cycles of satellite homogenization and the elimination of elements such as Athila, as observed in *Arabidopsis* (Naish et al., 2021; Bousios et al., 2025). Wlodzimierz et al. (2023) in his work proposes a different stage of satellite homogenization in *A. thaliana*, and compared to *A. lyrata* centromeres. The author proposes rapid cycles of transposon invasion and purging during satellite homogenization which bolsters the non-stochastic distribution of Cl0001 in our results.

### 4.3 oxi-mCs are preferentially observed at rDNA loci

Ribosomal DNA arrays are known to exhibit complex epigenetic regulation (Srivastava et al., 2016), with only a subset of copies being transcriptionally active at any given time. Such phenomenon is well described in *A*. *thaliana* which contains three major 45S rDNA variants, keeping only one of them active in germinating seeds (Pontvianne et al., 2010). In many plant species, inactive rDNA repeats are associated with heterochromatic marks, whereas active rDNA clusters display reduced methylation and distinct chromatin states (Bartova et al., 2010; Chandrasekhara et al., 2016; Kutashev et al., 2021; Fultz et al., 2023). In addition, the number of transcriptionally active rDNA units varies among cell lineages, which reflects effective on–off regulation through epigenetic mechanisms, chromatin condensation, and cell-cycle-dependent spatial organization (Neves et al., 2005). A major finding in this study is the strong association between oxi-mCs and 45S rDNA clusters. Mapping and feature coverage analyses revealed that nearly half of the rDNA copies across the genome were associated with at least one oxi-mC peak. Moreover, these peaks were concentrated primarily in highly expressed rDNA clusters located on chromosomes 2, 6 and 9 (**Figures 3**, **4**). The enrichment of oxi-mCs at rDNA loci was supported by multiple lines of evidence, including RepeatExplorer2 cluster annotation, sequence similarity analyses, and RNA expression levels. It is therefore tempting to speculate that 5caC is a half-mark of 45S rDNA subunit activation. ImmunoFISH experiments further demonstrated clear colocalization of 5fC and 5caC signals with active nucleolar organizing regions (NORs). Additionally, progressive accumulation of signals from 5hmC to 5fC and 5caC within the nucleolus indicates that these modifications may occur sequentially along the oxidative demethylation pathway which would be of support of 5caC peaks in expressed rDNA subunits. Our findings are consistent with the model of epigenetic regulation of rDNA, where active and inactive arrays are distinguished by DNA methylation and chromatin state (Simon et al., 2018; Kutashev et al., 2021). Our data suggests that oxidative cytosine derivatives may differentiate actively transcribed rDNA units, potentially reflecting ongoing DNA demethylation. This interpretation is supported by the enrichment of oxi-mCs specifically in highly expressed clusters and their nucleolar localization. These observations are consistent with those of Fultz et al. (2023) who associated hypomethylated regions with transcriptional activity in *Arabidopsis*.*is*. A work published by Cermak et al. (2008) identified a colocalization of the satellite TR1 and 45S rDNA arrays. The nucleolus is a nuclear structure without membrane but capable of keeping its own shape and can be physically isolated. It is the region for producing and assembling ribosomal subunits and the Nucleolar Organizing Regions (NORs) are part of the nucleolus (Ponti, 2025). The expressed copies are variants in structure and expression and the nucleolar organization is a proxy for transcription (Ramgopal et al., 2026), which it is also true for the satellite TR1. Several histone modifications have been described in the regulation of 45S rDNA and thus, oxi-mCs may represent an additional regulatory layer contributing to rDNA activity, possibly by facilitating a more open chromatin state required for RNA polymerase I transcription.

### 4.4 Functional implications and emerging model

While some satellite repeats such as STAR-C shows partial association with specific oxi-mCs, this relationship does not appear to be strictly linked to centromere identity. In contrast, rDNA clusters display a strong and reproducible enrichment of oxidative cytosine modifications, pointing to a potential role in regulating ribosomal gene activity. These findings highlight the complexity of all oxi-mC suggesting these marks do not have a unique role as it has been proposed for 5hmC (Yan et al., 2025). In large plant genomes, repetitive sequences constitute the majority of genomic DNA and can influence chromatin organization, gene regulation, and genome evolution. The presence of oxi-mCs within these regions makes clear that cytosine oxidation pathways may play broader roles in plant genome regulation than previously recognized. However, the reality is TET homologues have not been found in plants despite different tools have been developed (Iyer et al., 2009, 2013). Given the different chemistry used in DIP and immunoFISH, we do not consider both protocols oxidate the same region in the nuclei. On the other hand, an environmental regulation by oxidative stress might be another possibility already suggested (Chia et al., 2011).

### 4.5 Limitations and future directions

This study is based on low-pass sequencing, which provides a partial view of genome-wide repeat composition and limits quantitative comparisons between sexes and modifications. While RepeatExplorer2 enables robust repeat identification without a complete genome assembly, it may underestimate low-abundance elements. ChIP-seq Mapper reference-free validation strengthens reliability over assembly-dependent mapping. This reveals oxi-mC as repeat classifiers, filling gaps in plant epigenetics beyond 5mC. Low-depth sequencing limits absolute quantification, but immunoFISH congruence across sexes strengthens causality. We uncover an active 5mC oxidation axis in plant rDNA, suggesting oxi-mC as euchromatin sensors during rDNA active transcription. Future work integrating higher-coverage sequencing, chromatin profiling, and functional assays will be necessary to determine the mechanisms responsible for generating these modifications and to clarify their biological roles in plant chromatin dynamics.

## Data Availability

The datasets presented in this study can be found in online repositories. The names of the repository/repositories and accession number(s) can be found in the article/**Supplementary Material**.
