# Supplementary material for: Methylcytosine oxidation derivatives are preferentially associated with repetitive chromatin and mark active rDNA in Silene latifolia
Source: Front Plant Sci. 2026 Sep 22;17:1918439. doi: 10.3389/fpls.2026.1918439 (PMC13638503; doi:10.3389/fpls.2026.1918439)
Supplement: Supplementary file 1 [file DataSheet1.pdf]

Supplementary material

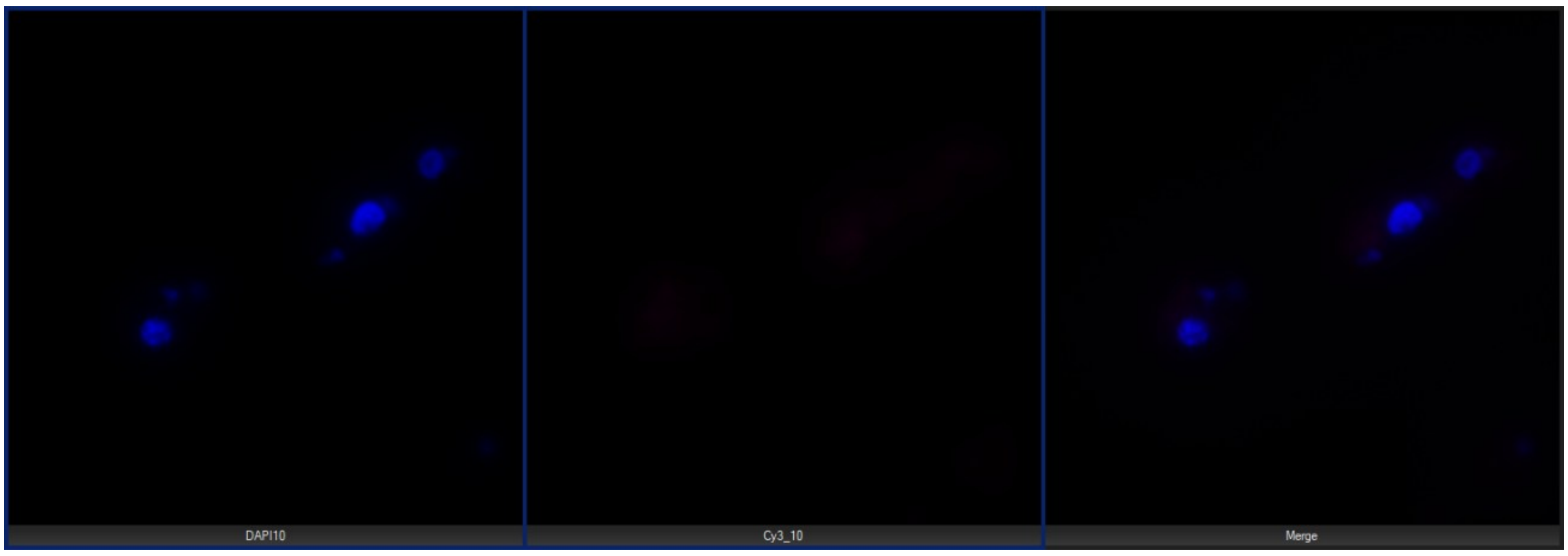

Supplementary Figure 1. Negative control in *S. latifolia* nuclei, individual and merged color channels are displayed. A secondary antibody incubation conjugated with Cy3 fluorescein (goat α-rabbit; AB_2313593) was performed without a primary antibody to test specificity. DAPI is shown in blue, a lack of antibody signal in red is observed.

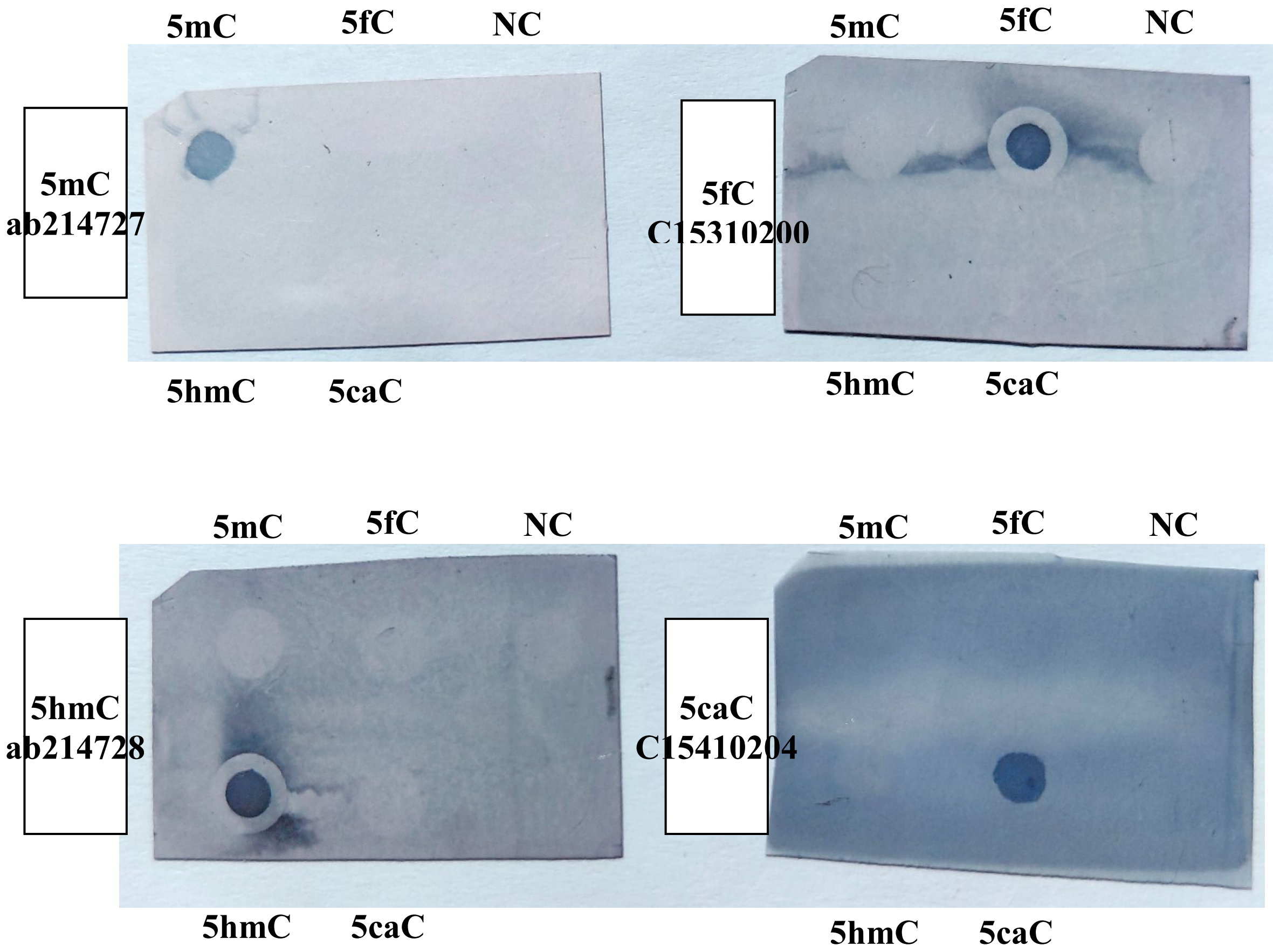

Supplementary Figure 2: Dot blot to test the cross-reactivity of the different antibodies, using the binary vector pCAMBIA-2301 to generate an amplicon of 389 bp. In the PCR, for each reaction, cytidine was replaced with the corresponding modified nucleoside and detected with the appropriate antibody: 5mC (Abcam, cat. no. ab214727), 5hmC (Abcam, cat. no. ab214728), 5fC (Diagenode, cat. no. C15310200), and 5caC (Diagenode, cat. no. C15410204-100). The same pCAMBIA-2301 probe with cytidine was used as negative control. A total of 10 ng of the corresponding purified product was spotted on the membrane and the detection was based on the AP-conjugated anti-rabbit IgG (Alcaline phosphatase) and the chromogenic substrate NBT/BCIP.

Supplementary Table 1. Significant clusters for CENH3 according to ChIP-seq Mapper using the repeat CENH3 cluster library from RepeatExplorer2. Main centrophilic repeats from *S. latifolia*; STAR-C (STAR-Y for Y chromosome) and CRM are represented in the table.

Cluster	Repeat	Chip_Hits	Input Hits	Ratio Chip/Input
9	STAR-C/STARY	42736569	1963818	21.76198
130	CRM	500028	47826	10.45515
59	CRM	1031547	103404	9.975891
172	CRM	166255	18115	9.177753

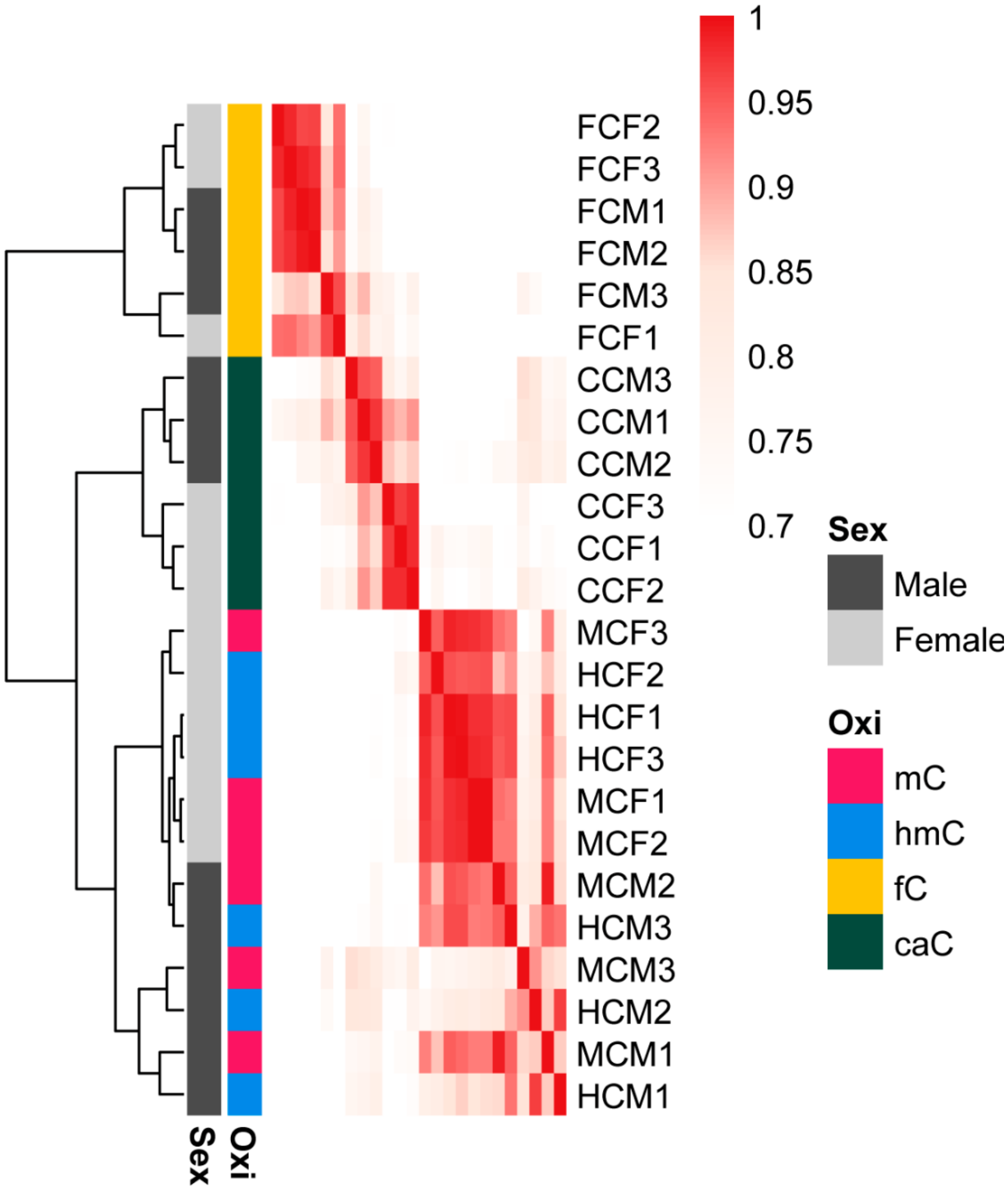

Supplementary Figure 3. Pearson correlation distance heatmap based on the ChIP-seq Mapper normalized ratio Chip/Input for each individual replicate against the *S. latifolia* gDNA based repetitive clustering library from RepeatExplorer2 clustering tool. The clustering evidences three main groups, one for 5fC, other for 5caC and a third group with strong correlation including 5mC and 5hmC.

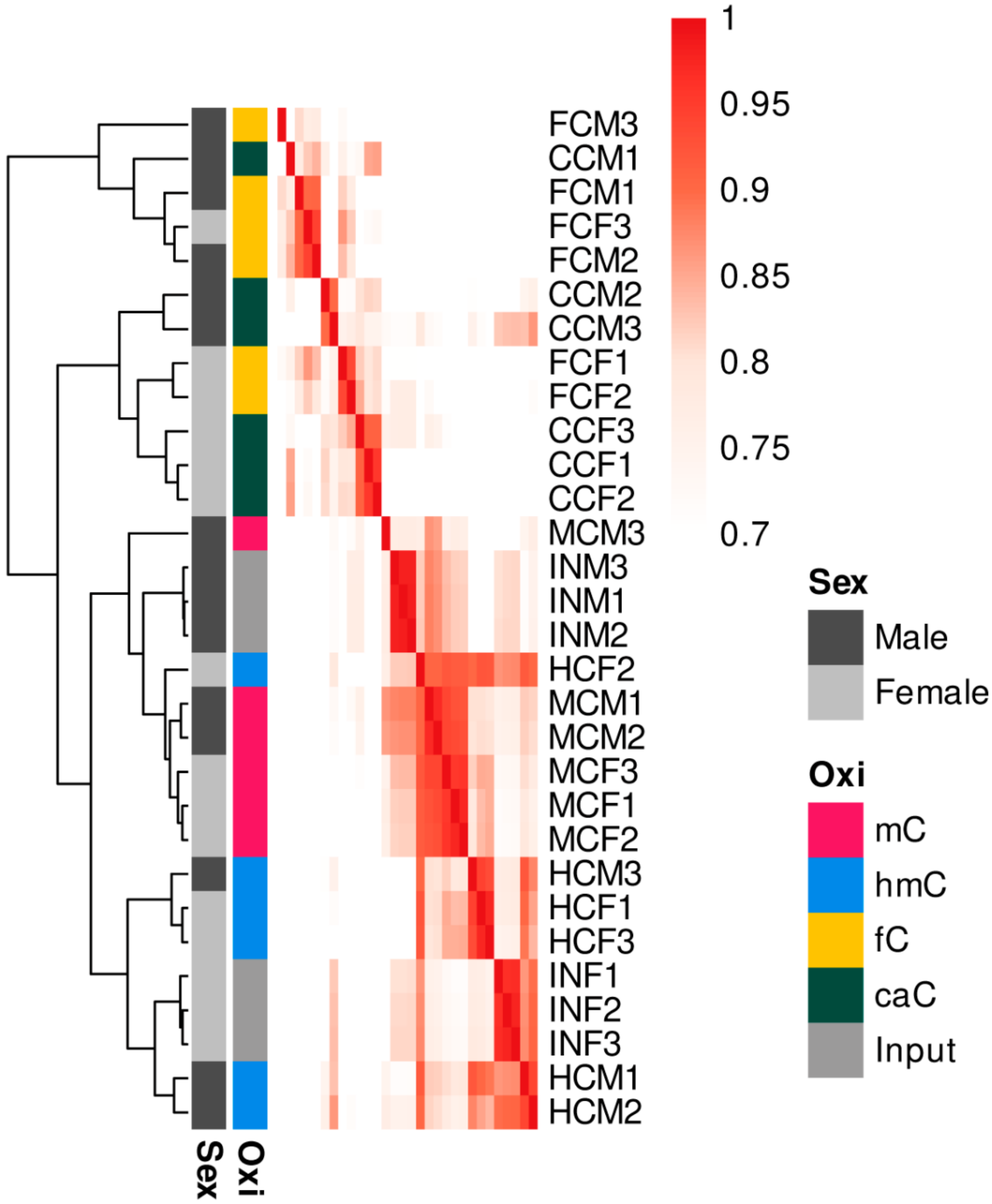

Supplementary Figure 4. Pearson correlation distance heatmap based on the BAM files after mapping the reads in the *S. latifolia* genome. The clustering evidences three main groups, one for 5fC, other for 5caC and a third group with strong correlation including 5mC and 5hmC. The clusters are not so clear as in Supplementary Figure 1, but 5fC and 5caC are clustered together compared to 5mC and 5hmC replicates and for each condition, at least two replicates cluster together.

Supplementary Table 2. Within-condition correlation coefficients among modifications and replicates on the BAM files after mapping the reads in the *S. latifolia* genome. No replicate showed a consistently discordant pattern with the other replicates from its condition.

Condition	Replicate correlations	Mean r
5caC female	0.957, 0.908, 0.909	**0.925**
5caC male	0.762, 0.694, 0.898	**0.785**
5fC female	0.938, 0.862, 0.821	**0.874**
5fC male	0.906, 0.811, 0.781	**0.832**
5hmC female	0.925, 0.975, 0.919	**0.940**
5hmC male	0.941, 0.921, 0.879	**0.914**
Input female	0.967, 0.969, 0.977	**0.971**
Input male	0.984, 0.981, 0.980	**0.982**
5mC female	0.976, 0.958, 0.959	**0.964**
5mC male	0.970, 0.865, 0.857	**0.897**

Supplementary Table 3. ChIP-seq Mapper enriched clusters using the *S. latifolia* repeat gDNA cluster library from RepeatExplorer2. The significant corrected clusters enriched for each of the four cytosine modifications are listed. The presence of the different cytosine modifications/sex combination on the right means enriched modification for the specific sex.

Cluster	Repeat	♂				♀			
4	LTR/unknown						5hmC		
6	LTR/unknown	5mC				5mC	5hmC		
7	LTR/unknown						5hmC		
9	STAR-C								5caC
21	LTR/unknown		5hmC				5hmC		
118	LTR/copia		5hmC				5hmC		
144	Helitron⇔Tekay						5hmC		
155	Simple repeats			5fC				5fC	
188	Helitron⇔LTR/unknown		5hmC	5fC		5mC	5hmC	5fC	
205	Simple repeats⇔LTR/unknown						5hmC		
206	Helitron⇔LTR/unknown						5hmC		
207	Helitron				5caC	5mC	5hmC		5caC
233	Athila	5mC	5hmC						
264	Helitron⇔LTR/unknown					5mC	5hmC		

Supplementary Table 4. ChIP-seq Mapper enriched clusters using the *S. latifolia* repeat oxi-mC cluster library from RepeatExplorer2. The significant corrected clusters enriched for each of the four cytosine modifications and sex are listed. The presence of the different cytosine modification/sex combination on the right means enriched modification for the specific sex. There is a wider distribution for all cytosine modifications and the same cluster is commonly significant for more than one modification. As an exception, the cluster corresponding to STAR-C is significant for 5caC exclusively.

Cluster	Repeat	♂				♀			
1	satellite/STAR-C								5caC
3	LTR/unknown					5mC	5hmC		
7	LTR/unknown					5mC	5hmC		
9	LTR/unknown	5mC				5mC	5hmC		
10	LTR/unknown					5mC	5hmC		
12	LTR/unknown			5fC	5caC			5fC	5caC
14	LTR/unknown	5mC	5hmC			5mC	5hmC		
25	Angela		5hmC			5mC	5hmC		
31	Tat	5mC	5hmC	5fC	5caC	5mC	5hmC	5fC	5caC
35	Athila	5mC	5hmC			5mC	5hmC		
57	Ogre		5hmC				5hmC		
66	LTR/unknown<=>Athila	5mC	5hmC	5fC		5mC		5fC	
77	Tat		5hmC						
94	LINE	5mC	5hmC			5mC	5hmC		
116	CRM	5mC	5hmC			5mC	5hmC		5caC
121	LTR/unknown<=>Ogre					5mC			
136	LTR/unknown		5hmC						
150	LTR/unknown	5mC	5hmC	5fC	5caC	5mC	5hmC	5fC	5caC
168	Simple repeats			5fC	5caC			5fC	5caC
176	Athila				5caC	5mC	5hmC		5caC
177	Simple repeats<=>Angela	5mC	5hmC	5fC	5caC			5fC	5caC
185	CRM<=>LTR/unknown	5mC	5hmC	5fC	5caC	5mC	5hmC	5fC	5caC
191	Simple repeats<=>LTR/unknown	5mC	5hmC	5fC	5caC	5mC	5hmC	5fC	5caC
193	LINE					5mC	5hmC		
195	LTR/unknown	5mC	5hmC	5fC	5caC	5mC	5hmC	5fC	5caC

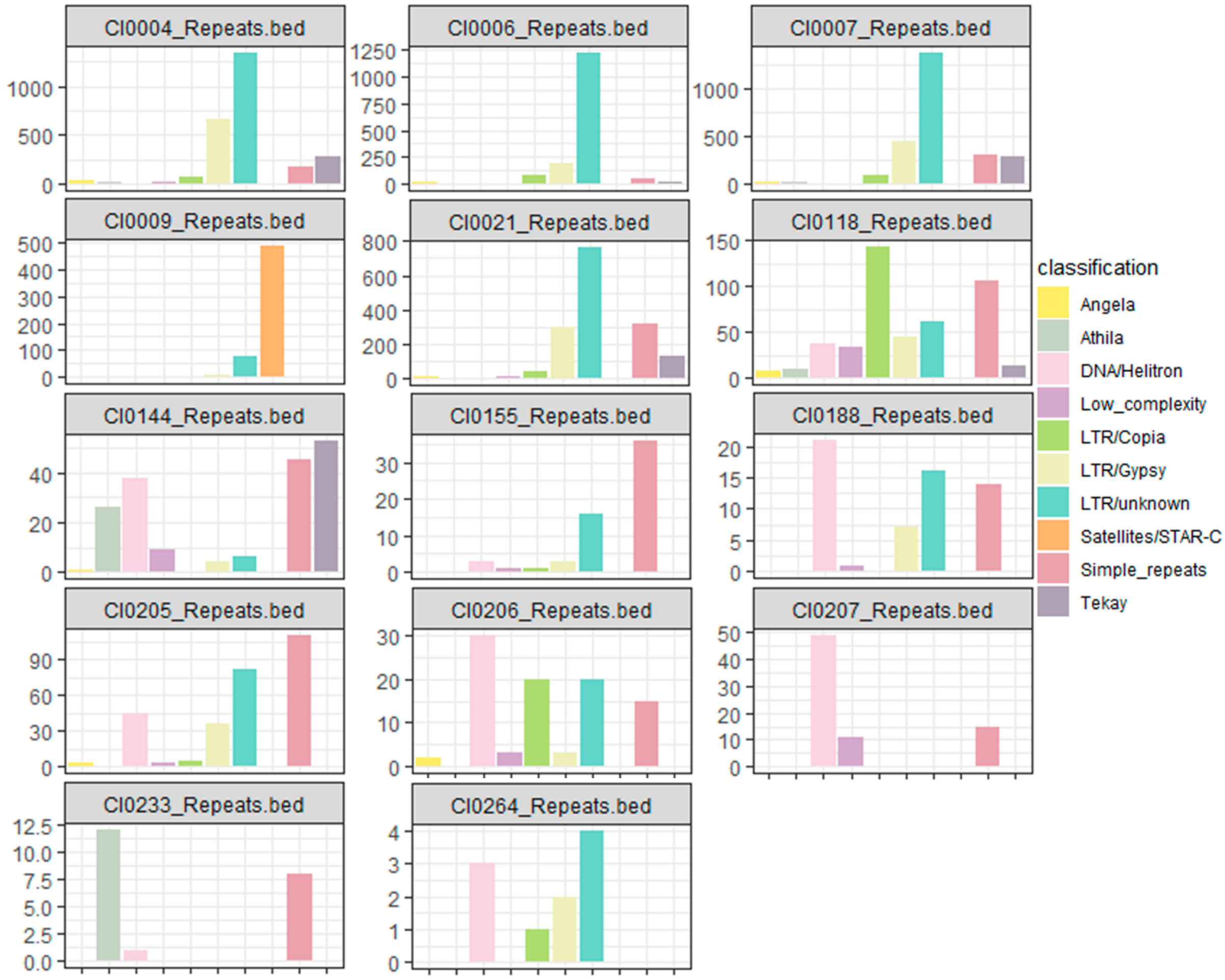

Supplementary Figure 5. Feature counts for each significant cluster from ChIP-seq Mapper using *S. latifolia* gDNA cluster library. Each cluster was annotated based on the feature counts hit according to the genome annotation from Moraga et al., 2025. A main repeat or a majority combination of few repeats is observable for each cluster.

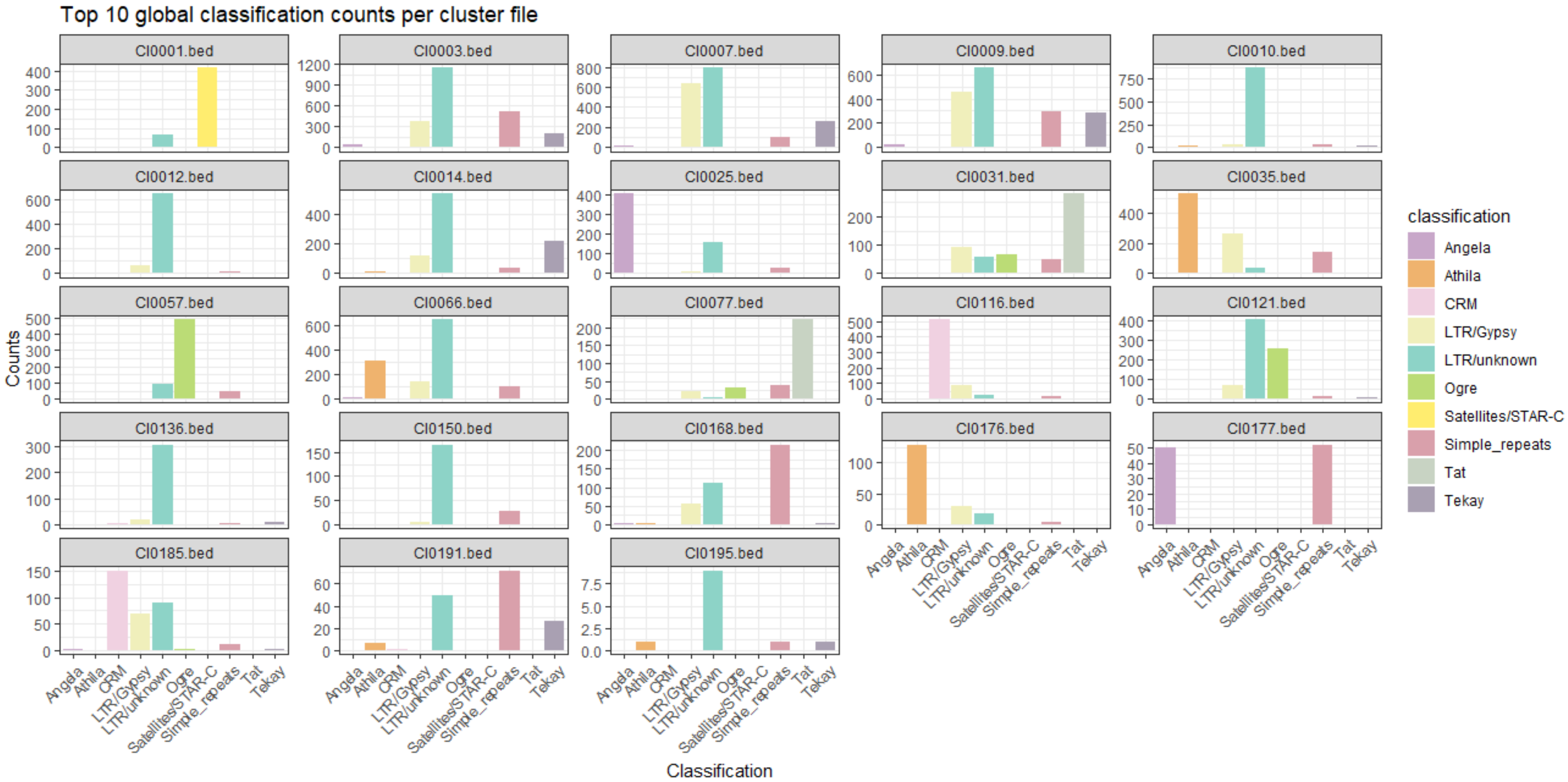

Supplementary Figure 6. Feature counts for each significant cluster from ChIP-seq Mapper using *S. latifolia* gDNA cluster library. Each cluster was annotated based on the feature counts hit according to the genome annotation from Moraga et al., 2025. A main repeat or a majority combination of few repeats is observable for each cluster.

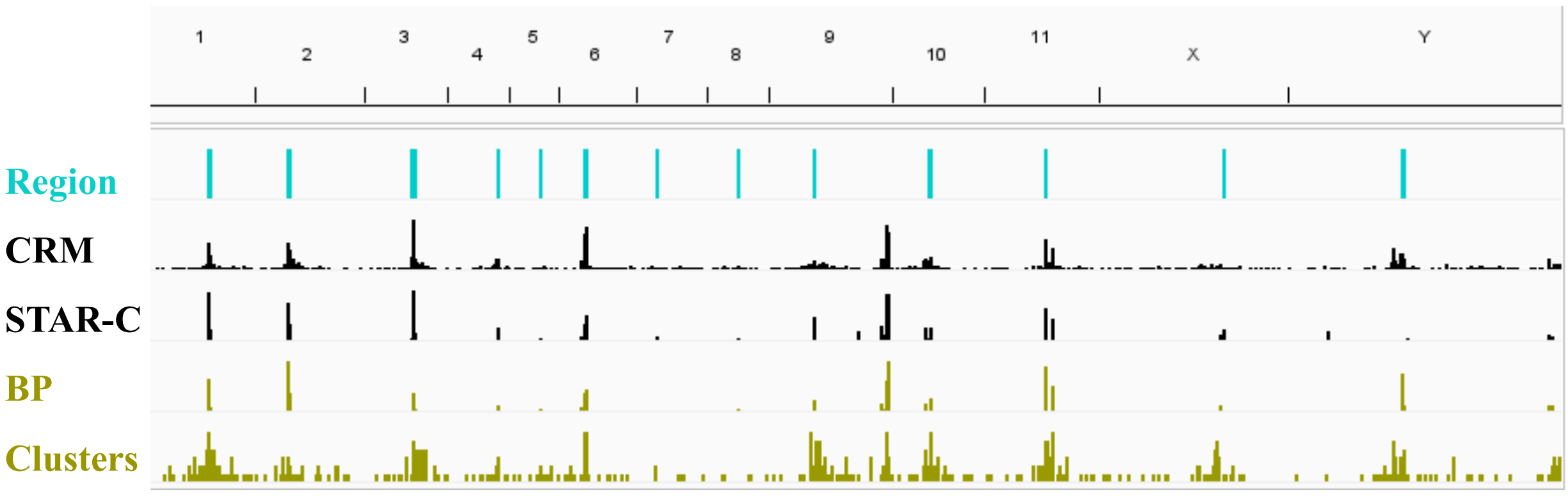

Supplementary Figure 7. Whole genome interval visualization from the different files used to determine the operational centromere regions in *S. latifolia*. Selected centromere region (turquoise blue), centromeric repeats CRM and STAR-C (black) from Moraga et al., 2025 and CENH3 broadpeaks (BP) and clusters (mustard green) from this study.

A

STAR-C Phylogenetic tree	
Parsimony score	23465
Permutation test (n 1000)	p < 0.001 ***
**STAR-C clustering**	
Mean chromomosome/cluster	0.997163
Median chromosome/cluster	1
Clusters with single chromosome	61250
Total clusters	61894
Permutation test (n 1000)	p < 0.001 ***

B

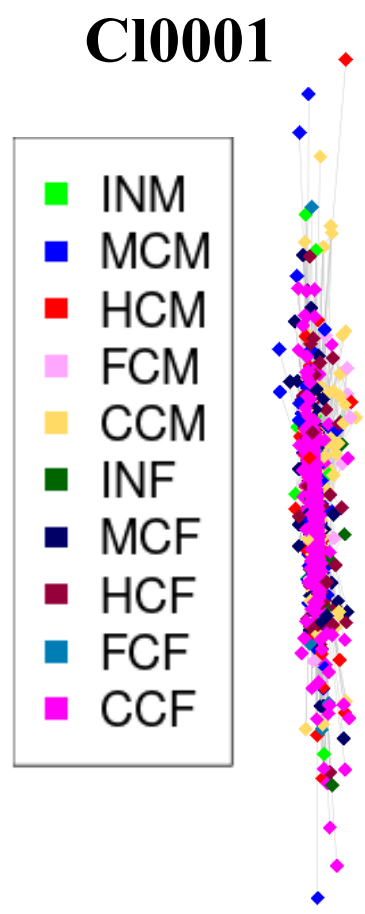

Supplementary Figure 8. A) Tests performed in the phylogenetic tree from the multiple sequence alignment for all the STAR-C features in *S. latifolia* genome according to Moraga et al., 2025. Chromosome combination in the clusters from all the STAR-C features in *S. latifolia* genome according to Moraga et al., 2025. Only those identical features (100 % identity) where filtered for the same cluster, more than half of the clusters are completely chromosome-specific. B) Representation of cluster Cl0001 corresponding to STAR-C according to RepeatExplorer2 comparative analysis. The highest signal corresponds to 5caC for both sexes. INM-Input male, MCM-5mC male, HCM-5hmC male, FCM-5fC male, CCM-5caC male, INF-Input female, MCF-5mC female, HCF-5hmC female, FCF-5fC female, CCF-5caC

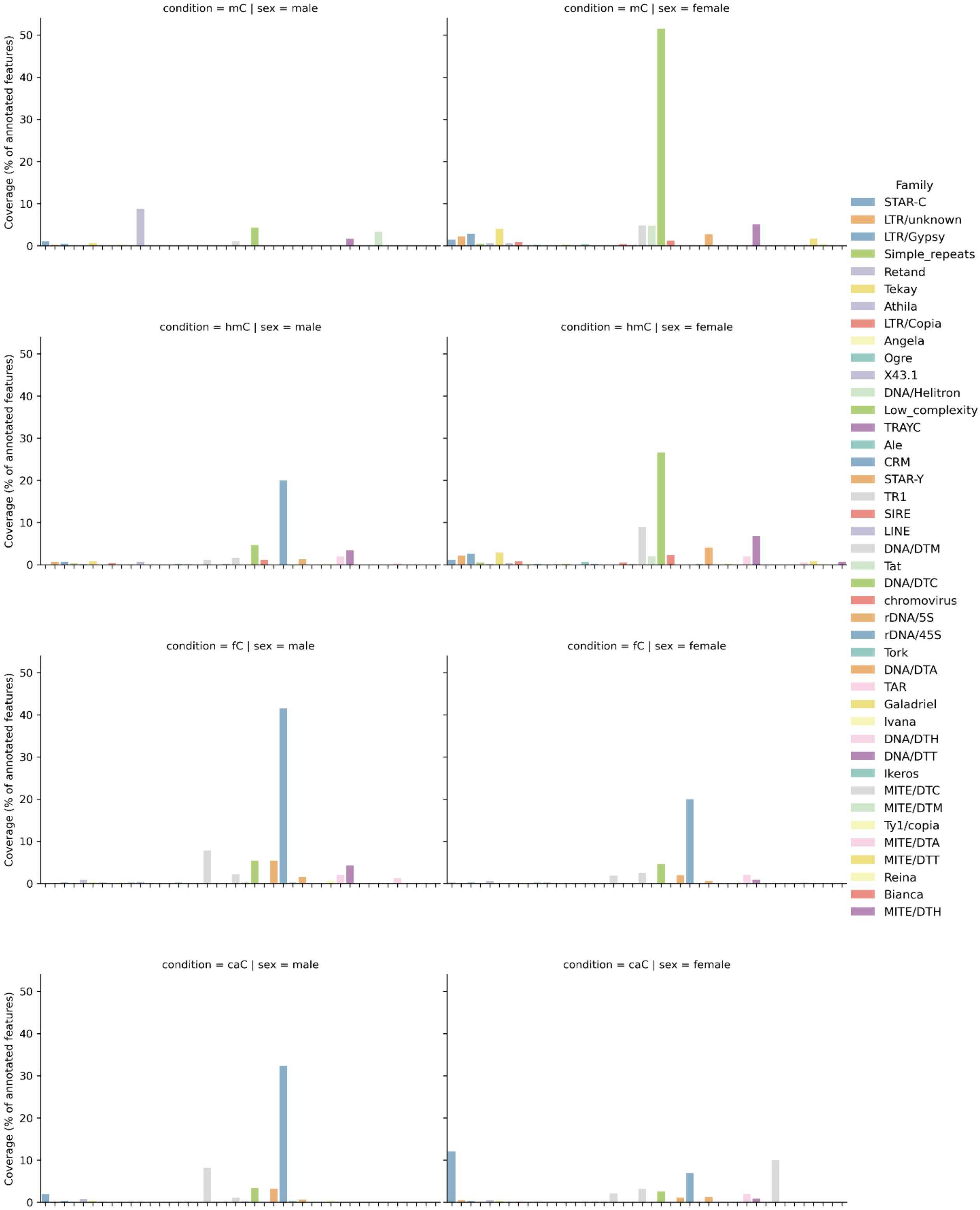

Supplementary Figure 9. Feature counts coverage percentage for each annotated repeat present in the *S. latifolia* genome based on the annotations from Moraga et al., 2025. A repeat, 45S rDNA, displayed a coverage around 20 % in most of the cytosine oxidative modifications.

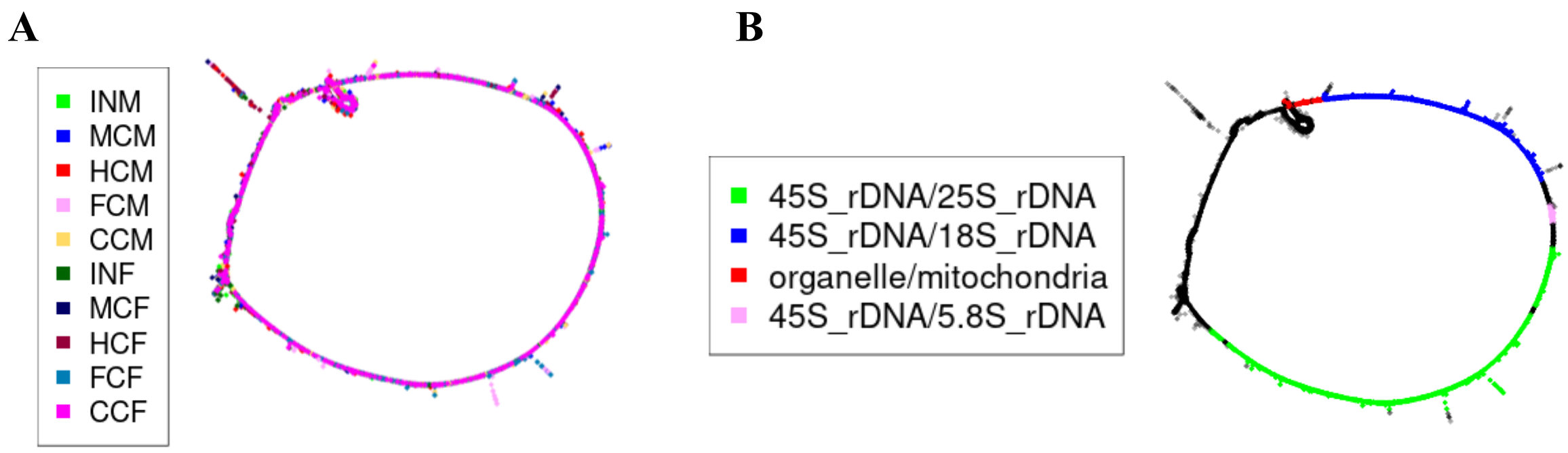

Supplementary Figure 10. A) RepeatExplorer2 comparative analysis of annotated cluster Cl0086 for Input and the different oxi-mC in male and female *S. latifolia*. B) Protein domain annotation of Cl0086 including the different subunits of 45S rDNA. The oxi-mC 5caC is the predominant mark covering the different subunits.

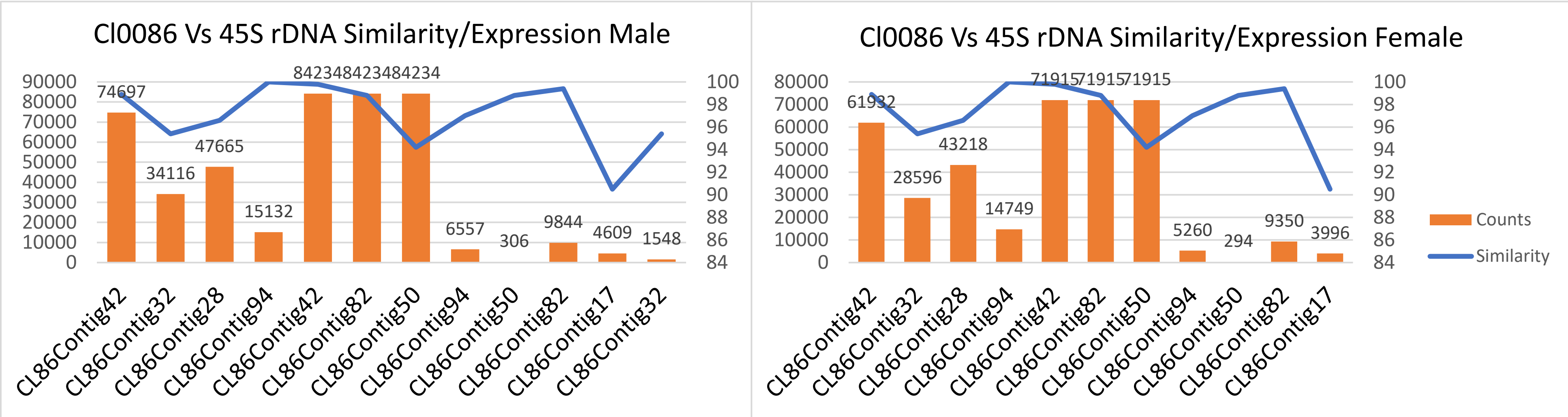

Supplementary Figure 11. Contigs from cluster Cl0086 with similarity over 90% compared to the sequence of the 115 features along the male and female genome of *S. latifolia* (blue line - right axis). More than one contig hit the same feature. The feature intervals with similarity over 90% belonged to chromosomes 2, 6, 9 and Y for males. The expression in raw counts (orange bars – left axis) is represented for each interval. A high number of raw counts is observed for most intervals.

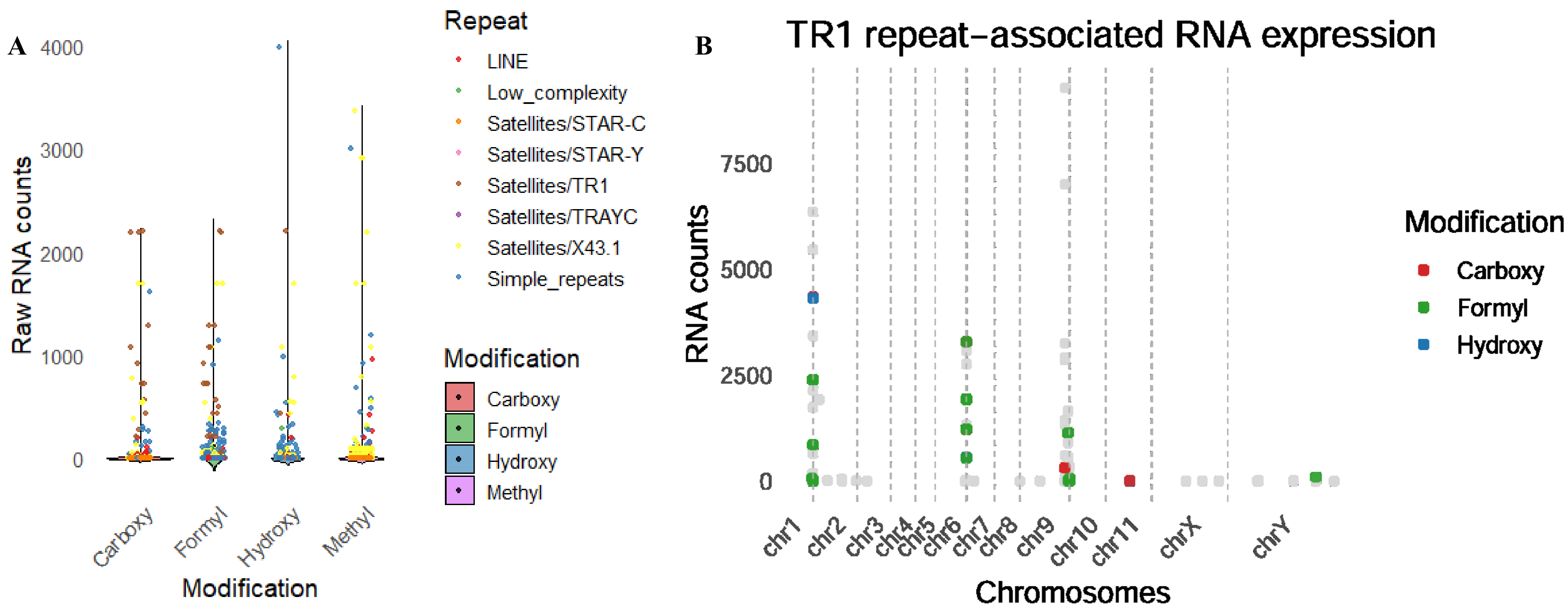

Supplementary Figure 12. A) Distribution of raw mRNA counts in satellites and tandem repeats excluding ribosomal DNA. Each dot corresponds to a unique feature covered by at least one summit from the oxi-mC identified in this work. A few features displayed hundreds or thousands raw counts. One family of satellites TR1 has a large number of features with high mRNA counts. B) Scatter plot mapping of satellite TR1 feature expression intersected with oxi-mC summits along *S. latifolia* chromosomes. Colored dots based on different oxi-mCs and sex mainly gather around chromosomes 2 and 9, and in a lesser extent in chromosomes 1 and 6. These regions display highly expressed copies and match with regions where active 45S rDNA clusters are present.

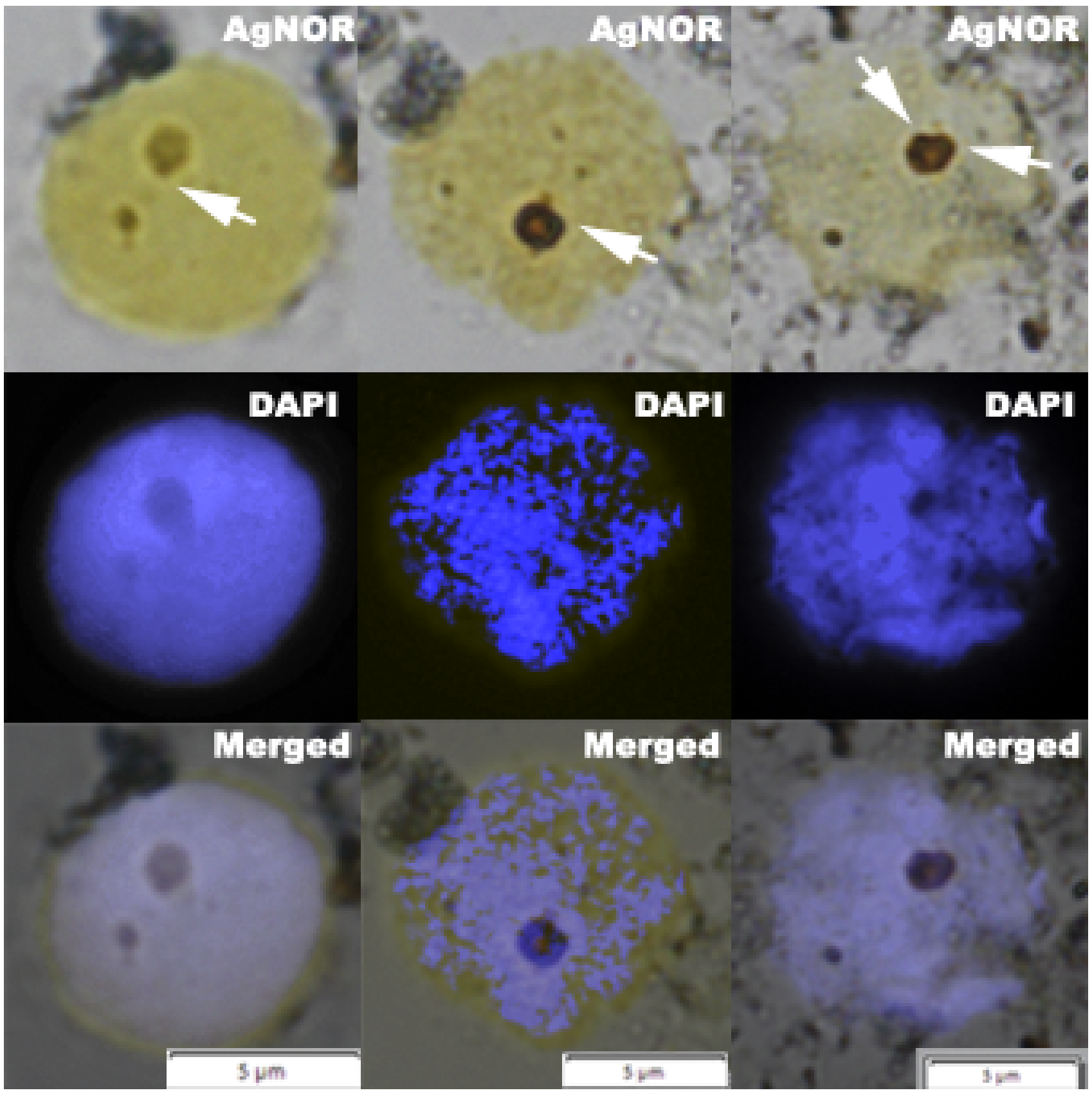

Supplementary Figure 13. Nucleolar Organizing Region stained with silver nitrate (AgNOR) on *S. latifolia* nuclei. White arrows indicate the active rDNA 45S clusters as darker dots more sensitive to silver nitrate present in the NOR. Bar 5 µm

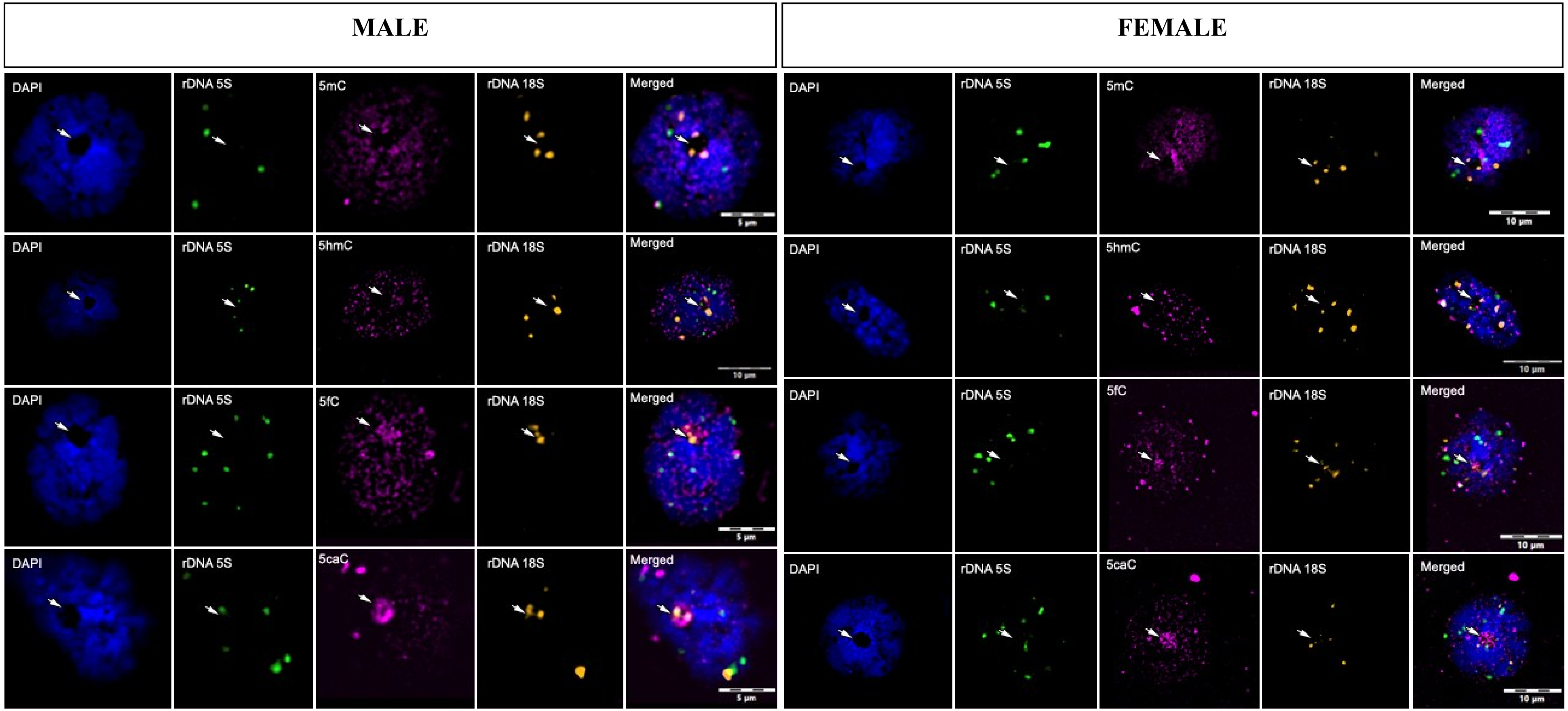

Supplementary Figure 14. ImmunoFISH on male and female *S. latifolia* nuclei, individual and merged color channels are displayed. An antibody signal against 5caC is shown in magenta, a probe for rDNA 45S is shown in yellow, a probe for rDNA 5S is shown in green and DAPI is shown in blue. The white arrow points to the nucleolar organizing region. An increasing signal concentration for the progressive cytosine oxidatives is observed in the nucleolus. The two active rDNA 45S clusters present in the nucleolus overlap their signal with 5fC and 5caC signal.

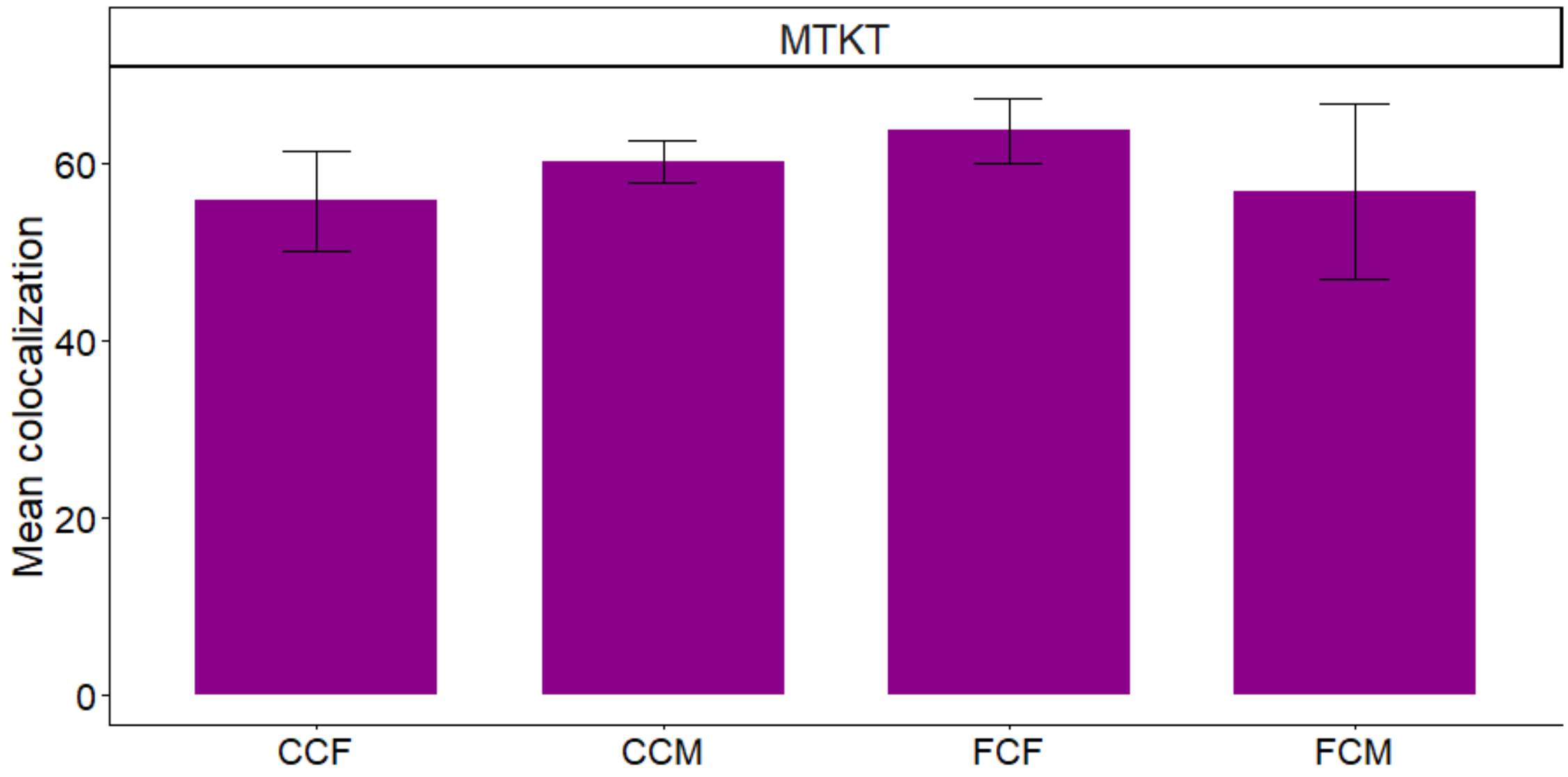

Supplementary Figure 15. Mean colocalization according to Maximum Truncated Kendall Tau between 5fC and 5caC signal and active rDNA 45S clusters. In all situations a high, positive, non-random colocalization correlation was observed. (n = 6)
